# Kratom Alkaloids: A Blood–Brain Barrier Specific Membrane Permeability Assay-Guided Isolation and Cyclodextrin Complexation Study

**DOI:** 10.3390/molecules29225302

**Published:** 2024-11-09

**Authors:** András Dohárszky, Erika Mária Vági, Árpád Könczöl, Alexandra Simon, Erzsébet Várnagy, Miras Muratov, Kristóf István Steiger, Bianka Várnai, Szabolcs Béni, Eszter Riethmüller, Ida Fejős

**Affiliations:** 1 Department of Pharmacognosy, Semmelweis University, Üllői út 26, H-1085 Budapest, Hungary; doharszky.andras@stud.semmelweis.hu (A.D.); konczol.capice@gmail.com (Á.K.); simon.alexandra1@semmelweis.hu (A.S.); varnagy.erzsebet@phd.semmelweis.hu (E.V.); stofi1998@gmail.com (K.I.S.); varnai.bianka@phd.semmelweis.hu (B.V.); szabolcs.beni@ttk.elte.hu (S.B.); 2 Center for Pharmacology and Drug Research & Development, Semmelweis University, H-1085 Budapest, Hungary; 3 Department of Chemical and Environmental Process Engineering, Faculty of Chemical Technology and Biotechnology, Budapest University of Technology and Economics, Műegyetem rkp. 3, H-1111 Budapest, Hungary; vagierikamaria@gmail.com (E.M.V.); mirasmuratov1998@gmail.com (M.M.); 4 Integrative Health and Environmental Analysis Research Laboratory, Department of Analytical Chemistry, Institute of Chemistry, ELTE Eötvös Loránd University, Pázmány Péter sétány 1/A, H-1117 Budapest, Hungary

**Keywords:** indole alkaloids, extraction, parallel artificial permeability assay, blood–brain barrier permeability, inclusion complexes, complex stability, bioavailability enhancement, affinity capillary electrophoresis, phase-solubility study, *Mitragyna speciosa*

## Abstract

Mitragynine is an “atypic opioid” analgesic with an alternative mechanism of action and a favorable side-effect profile. Our aim was to optimize the alkaloid extraction procedure from kratom leaves and to determine and isolate the most relevant compounds capable of penetrating the central nervous system. The PAMPA-BBB study revealed that mitragynine and its coalkaloids, speciociliatine, speciogynine, and paynantheine, possess excellent in vitro BBB permeability. An optimized sequence of CPC, flash chromatography, and preparative HPLC methods was used to isolate the four identified BBB+ alkaloids. To improve the bioavailability of the isolated alkaloids, their cyclodextrin (CD) complexation behavior was investigated via affinity capillary electrophoresis using almost 40 CD derivatives. The apparent alkaloid–CD complex stability constants were determined and compared, and the most relevant CDs phase-solubility studies were also performed. Both the neutral and negatively charged derivatives were able to form complexes with all four kratom alkaloids. It was found that cavity size, substituent type, and degree of substitution also influenced complex formation. The negatively charged Sugammadex, Subetadex, and the sufoalkylated-beta-CD analogs were able to form the most stable complexes, exceeding 1000 M^−1^. These results serve as a good basis for further solubility and stability enhancement studies of kratom alkaloids.

## 1. Introduction

Kratom (*Mitragyna speciosa* Korth., Rubiaceae) is a psychoactive evergreen tree native to Southeast Asia [1]. Kratom leaves have been traditionally used for centuries for pain relief and wound healing or consumed as a recreational drug due to their stimulant effect [2]. In addition, kratom leaves are used as a substitute for opium or to treat opioid withdrawal symptoms [3]. Corynanthe-type monoterpene indole alkaloids, particularly the chief alkaloid mitragynine, are responsible for kratom’s therapeutic effects. Mitragynine binds to opioid receptors, especially the µ-opioid receptor (MOR). However, unlike the classical opioids such as morphine and heroin, mitragynine’s binding activates the G-protein-coupled signaling cascade without triggering β-arrestin-2 activation. The activation of β-arrestin-2 is associated with the adverse effects of opioid receptor activation, i.e., respiratory depression, constipation, and addiction [4,5]. Thus, this “atypical opioid agonist” has therapeutic potential for pain management with limited adverse effects compared to classical opioids. The use of mitragynine as an analgesic has been proven through comprehensive preclinical and clinical studies [6]. The apparent low efficacy of mitragynine at µ-opioid receptors supports the development of these ligands as effective and potentially safe medications for opioid use disorder [7].

Opioid receptors are found throughout the body, including the intestinal tract and the central nervous system (CNS). MORs are the most frequently expressed opioid receptors, which are predominantly present in the CNS [8]. In order to bind to the central MORs and to achieve CNS effects, substances must cross the blood–brain barrier (BBB) first. It is therefore important to clarify which components of kratom are able to enter the central nervous system (BBB+). The parallel artificial permeability assay (PAMPA) provides a rapid and simple in vitro method of investigating the permeability of single molecules, as well as multicomponent plant extracts. Moreover, PAMPA experiments coupled with a suitable HPLC method may help to explore the most relevant BBB+ components of the extracts; thus, PAMPA could govern the isolation procedure of promising plant metabolites, which may contribute to the CNS effects.

However, mitragynine shows intermediate lipophilicity (log*P* = 1.70, log*D* = 1.73 at pH 7 [9]) and moderate aqueous solubility (at neutral pH 88 mg/L [9]), and it is considered unstable under acidic conditions (with more than 26% degraded after 2 h [10]), limiting its potential clinical use as an analgesic agent [11]. The limited oral bioavailability of mitragynine was also reported in rats [12]. Cyclodextrins (CDs) are excellent tools for improving bioavailability, as the polar nature of the outer surface of CDs ensures their good aqueous solubility, while their weakly hydrophobic inner cavity can accommodate apolar guest molecules through inclusion complex formation. A crucial point of the optimization of the pharmacokinetic properties and bioavailability of mitragynine and its coalkaloids is the selection of the appropriate CD derivative. The CD complex formation ability of mitragynine with native-β-CD and sulfobutylated-β-CD was studied by our research group [13]. To complete these experiments and to compare a wide spectrum of CDs, environmentally friendly, low-cost, and low-sample-consuming affinity capillary electrophoresis (ACE) method provides great opportunity [14,15].

In this work, our aim was to find the most relevant kratom alkaloids capable of penetrating the blood–brain barrier and to enhance their bioavailability by applying CD derivatives as complexing agents. Based on the results of PAMPA-BBB studies, we planned the isolation of BBB+ compounds using centrifugal partition chromatography (CPC), flash chromatography, and preparative HPLC. In order to study the CD complexation ability of the isolated kratom alkaloids, we aimed to develop an ACE method capable of screening a wide range of variously substituted CD derivatives. Phase-solubility studies were conducted to demonstrate the CD-induced improvement in the water solubility of mitragynine.

## 2. Results and Discussion

### 2.1. Extraction Studies

To produce alkaloid-enriched extracts, different extraction methods were studied in a comprehensive way. Traditional maceration and Soxhlet extraction using different solvents (ethanol, methanol, and methanol acidified with HCl) were studied with laboratory-scale apparatuses, while the efficiency of ultrasound-assisted extraction (UAE) and supercritical fluid extraction (SFE) with a carbon dioxide and an ethanol cosolvent at the pilot scale was also investigated.

Several qualitative and quantitative analytical methods have been reported for the investigation of herbal or biological samples containing mitragynine or other kratom alkaloids [16]. Our aim was to develop an UHPLC-UV method capable of monitoring the extraction procedures, to analyze the samples of the later-discussed PAMPA-BBB test, to check the purity of the isolated compounds, and to assess the solubility enhancement of mitragynine in the phase-solubility studies. The optimized UHPLC-UV method was validated according to the ICH guidelines [17] for specificity, selectivity, linearity, limit of detection (LOD), limit of quantitation (LOQ), precision, and accuracy (see Table S1 in the Supplementary Materials). A successful separation of the four alkaloids was achieved with the optimized method, and no interfering peaks were detected in the obtained chromatograms. The method provided linear responses (r^2^ > 0.999) within the investigated range for mitragynine, speciogynine, speciociliatine, and paynantheine. The LOD parameter was in the 0.04–0.16 mM range, and the LOQ was between 0.11 and 0.48 mM for the four alkaloids. For more detailed validation parameters, see Table S1 in the Supplementary Materials.

**Figure 1:**
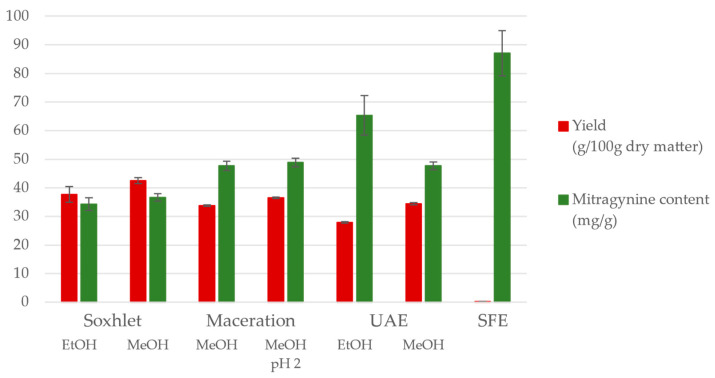
Comparison of the extraction procedures (Soxhlet extraction, maceration, ultrasound-assisted extraction (UAE), and supercritical CO_2_ extraction (SFE)) applying methanol (MeOH) and ethanol (EtOH) regarding the yield and the mitragynine content of the extracts. Detailed results and further conditions can be found in Table S2 (in the Supplementary Materials) and in Section 3.2.

The extraction yields and mitragynine contents of the variously prepared extracts are demonstrated in Figure 1 and summarized in Table S2 in the Supplementary Materials. With Soxhlet extraction, high extraction yields were achieved with ethanol, 37.7 ± 2.8% (g/100 g dry matter, d.m.); with methanol, the average yield was higher at 42.5 ± 1.0%. The samples contained the lowest amounts of mitragynine, 34.3 and 36.7 mg/g, likely due to the prolonged extraction process at the boiling point of the solvents. In a previous study, Harizal et al. employed methanolic Soxhlet extraction to produce a standardized *M. speciosa* extract, yielding 20 g of extract from 100 g of plant material, with a mitragynine content of 16 mg/g [18]. We found that the extraction yield could be twice that reported in the literature, while the mitragynine content was similar in our case (12.9–15.5 mg/g). The extraction yield depends largely on the particle size of the raw material, which might have caused the large differences in yield.

From the industrial point of view, the efficiency of simple maceration using methanol and acidified methanol (pH 2) solvents was further investigated. At 50 °C, the effect of extraction time was studied by decreasing the extraction time from 3 h to 30 min and then to 10 min. Very slight decreases in the extraction yields were observed (see Table S2 in the Supplementary Materials). The effect of extraction temperature was also examined, and there was an increase in the extraction yield from 32.0 ± 0.4% at 40 °C to 33.8 ± 0.3% at 50 °C for the shortest extraction time (10 min) investigated. When the acidity of the solvent was adjusted to pH 2, the highest extraction yield (36.5%) was achieved: a high mitragynine content of 48.9 ± 1.5 mg in 1 g of extract. The mitragynine contents in the extracts obtained by maceration using methanol solvent were between 47.6 ± 1.6 mg/g and 54.4 ± 3.4 mg/g, which was the highest measured alkaloid content in the extract obtained by maceration using methanol in a short 10 min extraction at 40 °C. However, the average yield was lower at this setting (30.5 ± 0.2%); still, the extract contained at least 1.7% (g/100 g d.m.) of mitragynine.

Furthermore, an innovative and environmentally benign technology, ultrasound-assisted extraction (UAE), was optimized. A 10 min extraction procedure was applied using methanol at different temperatures with different feed-to-solvent ratios (1:5, 1:10, and 1:15 g/L). The extraction yields and mitragynine contents in the extracts obtained with the different feed-to-solvent ratios were not significantly different (see Table S2 in the Supplementary Materials). The effect of temperature was slightly more predominant: increasing the extraction temperature from 30 to 50 °C resulted in a 3% increase in the yield (from 30.9 ± 0.3% to 34.1 ± 0.3%), while the alkaloid contents were almost the same in the extracts. The most dramatic enhancement in the alkaloid content was observed when changing the extraction solvent to ethanol; however, yields were reduced (see Figure 1 and Table S2 in the Supplementary Materials). Haziqah et al. optimized extraction yield via the UAE of freeze-dried kratom tea with methanol. They achieved a maximum yield of 49.7% under conditions of 34 °C, a 25 min extraction time, and a drug-to-solvent ratio of 50 g:166 mL [19]. Kikura-Hanajiri et al. also examined optimal UAE conditions by varying the solvent (methanol, ethanol, acetonitrile) and extraction time (1, 3, 6 h). They used 10–50 mg of *M. speciosa* plant powder with 10 mL of solvent, determining that the most effective method was extraction with 80% methanolic aqueous solution at room temperature for one hour, followed by an overnight rest at room temperature. The mitragynine content of the extracts was 20 mg/g [20]. Our findings correlate well with the results reported in several papers. The extraction yield reported by Haziqah et al. was higher; however, no information was given on the mitragynine content of the extract; Kikura-Hanajiri et al. obtained an extract with a lower mitragynine content than our extract. Our optimized UAE method produced a twice-as-high alkaloid content with a 30–36% extraction yield using methanol as the solvent at a low feed-to-solvent ratio (1:10) and with a very short extraction time (10 min) at 50 °C.

High-pressure supercritical CO_2_ extraction with a 10% ethanol cosolvent was also applied to obtain an alkaloid-rich fraction from ground kratom. The extraction parameters were a 30 MPa pressure and a 40 °C temperature, and the extracts were collected as an ethanol solution due to the application of an ethanol cosolvent. After the evaporation of the solvent, dark-green sticky fractions were recovered and collected in a timely manner during the extraction. It can be seen from Figure 1 and Table S2 (in the Supplementary Materials) that the overall yield was relatively low (5.2%); however, the mitragynine contents of one-third of the samples collected after 2 h of extraction (using 50 kg CO_2_/kg kratom) were exceptionally high, exceeding 50–60 mg/g. Unfortunately, even though these fractions were very rich in alkaloids, their yields were very low and available only in very small quantities. According to the literature, SFE-CO_2_ methods typically result in yields between 0.3% and 0.9%, and the mitragynine content is less than 2 mg/g in the extract by applying the following parameters: 243 g of leaf powder was extracted using 28.8% EtOH as a cosolvent at 65 °C, 300 bar, 12 kg/h of CO_2_ flow, for 45 min [21]. Tohar et al. stated that the highest extraction yield, 1.56%, was achieved at 5000 psi (34.5 MPa) and 40 °C using a 60% ethanol modifier. In the first step, when pure CO_2_ was applied under the same pressure (5000 psi) but at a temperature of 60 °C, the yield was 1.36% [22].

**Figure 2:**
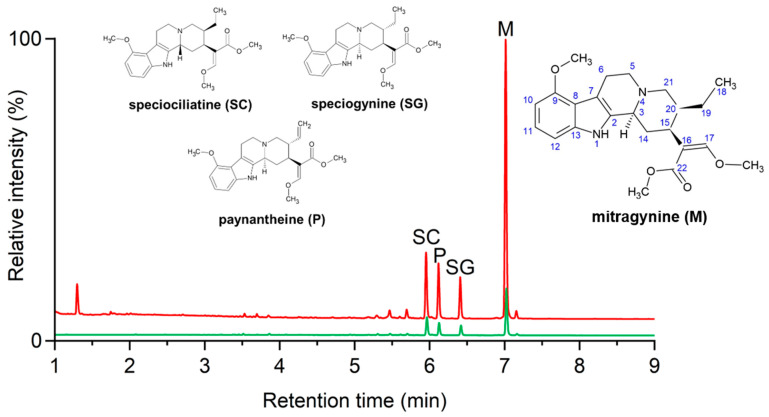
Demonstrative UHPLC-UV chromatogram of UAE MeOH (50 °C, 1:10 feed to solvent ratio) extract (red trace, top) and of PAMPA-BBB acceptor phase (green trace, bottom), along with the chemical structure of the kratom alkaloids detected in the acceptor phase recorded at 226 nm.

In conclusion, the various extraction methods studied, including Soxhlet extraction, maceration, ultrasound-assisted extraction, and SFE, demonstrated different levels of efficiency in yielding alkaloid-rich extracts (see Figure 1). However, the alkaloid profile did not vary notably with the extraction method: based on the UHPLC-UV experiments, mitragynine was the most abundant compound in all the extracts, followed by speciociliatine, paynantheine, and speciogynine. For the typical coalkaloid contents of the extracts, see the UAE results in Table S3 in the Supplementary Materials. The demonstrative chromatogram of the UAE extract (applying methanol, 50 °C, 1:10 feed to solvent ratio) is depicted in Figure 2 (red, top).

Comparing the used extraction procedures, Soxhlet extraction, particularly with methanol, yielded the highest overall extract quantities, though with a lower mitragynine content. UAE proved to be the most efficient for obtaining a high alkaloid content in a short time, while SFE, despite the lower overall yields, produced fractions with exceptionally high mitragynine concentrations. These findings highlight the importance of optimizing extraction parameters based on the specific yield and alkaloid concentration goals.

### 2.2. PAMPA-BBB-Guided Isolation Studies

Kratom extracts were tested for the membrane permeability of their constituents in a PAMPA-BBB assay. The PAMPA is one of the most effective screening tools used in early drug discovery to study passive transcellular transport. Mitragynine and a *M. speciosa* alkaloid extract previously demonstrated good permeability in a gastrointestinal PAMPA model at pH 7.4 (log*P_e_* − 4.95 ± 0.03 and −4.93 ± 0.03, respectively) [23]. The blood–brain barrier-specific assay was tested and optimized for natural products by Könczöl et al. [24]; this PAMPA-BBB assay was therefore chosen to determine the BBB permeability of the kratom constituents. Four main compounds were detected in the acceptor phase of the PAMPA model (see Figure 2 for demonstrative chromatograms), suggesting that they are able to cross the BBB via passive diffusion (BBB+). These results helped us to focus on the most relevant kratom components present in the highest concentrations in the extracts and supported the design of the isolation procedure. It should be noted, however, that among the minor components that were not the focus of this study, there may be BBB+ compounds with significant biological activity.

Isolation from the prepared kratom extracts posed considerable challenges due to the high ballast content, so we refrained from processing them and instead obtained a preprocessed extract. This extract was separated using a pilot-scale centrifugal partition chromatography (rCPC) procedure, yielding eight fractions labeled a–h. Since the BBB+ components were present in the highest proportions in fractions c and e, further isolation procedures via flash chromatography and semipreparative HPLC were performed on these fractions. The isolated compounds were identified as mitragynine, paynantheine, speciociliatine, and speciogynine via NMR spectroscopy (for the chemical structures, see Figure 2, NMR spectra are presented in the Supplementary Material, Figures S1–14).

The ^1^H and ^13^C NMR resonances of the isolated mitragynine were consistent with previously reported data [25]. For the two diastereomers of mitragynine (speciociliatine and speciogynine), key NMR characteristics were identified. Eight sp^2^ carbon atoms (C2 and C7–13) were assigned to the indole moiety. In the aromatic region of the ^1^H NMR spectrum, two doublets (H10 and H12) and a triplet (H11) were detected, indicating the presence of a trisubstituted phenyl ring (see Figures S4 and S8 in the Supplementary Materials). The signal corresponding to the vinylic proton at C17 and the methyl group at the C18 position were also identified (Table S4 in the Supplementary Materials). Additionally, the presence of three methoxy groups, attached to C9, C17 and C22, were observed in the ^1^H NMR spectrum (Table S4); these could also serve as selective resonances to establish qNMR studies [26]. Speciociliatine is the C3 epimer of mitragynine. The spatial orientation of H3 can be determined based on NOESY spectra. For mitragynine, the intramolecular NOE cross-peak between the H3 and H15 protons indicates the α orientation of H3. Therefore, the absence of this NOE cross-peak suggests a β orientation of H3. In the NOESY spectrum (Figure S7 in the Supplementary Materials), no such cross-peaks were identified, confirming the structure as speciociliatine. Speciogynine is the C20 epimer of mitragynine. The absence of the NOE cross-peak between H15 and H20 (see Figure S11) suggests that the orientation of H20 is the opposite to that in mitragynine (α), indicating a β orientation in the given molecule. Paynantheine exhibits the characteristic signals of mitragynine diastereomers previously highlighted. The NMR characteristics of paynantheine closely resemble those of speciogynine, with the main difference being the absence of signals corresponding to the ethyl moiety at position C20, which is replaced with a vinyl group. This is evidenced by two broad H18 doublets (*δ*_H_ 5.10 and 5.05 ppm) and an H19 multiplet (5.53 ppm), linked with a double bond Δ18–19 (*δ*_C_ 117.7 ppm and 136.1 ppm). The measured NMR spectra for all the isolated compounds (Figures S1–S14) and the key characteristic resonance assignments (Table S4) can be found in the Supplementary Material.

All four isolated kratom alkaloids were also tested in the PAMPA-BBB model. As it can be seen in Table 1, all the investigated compounds had log*P_e_* values of between −4.42 and −4.77, which were greater than −6.0; according to Könczöl et al. [24], this value effectively discriminates between compounds with good (BBB+) and poor (BBB−) blood–brain barrier permeability. Therefore, our results indicate that all major kratom alkaloids are capable of crossing the BBB by passive diffusion in vitro.

The main alkaloid, mitragynine, is the most widely studied component of kratom [9]; its excellent blood–brain barrier permeability has already been reported using the in vivo microdialysis technique [27]. Our PAMPA-BBB results are in good agreement with those of that study, while the expected good permeabilities for speciogynine, speciociliatine, and paynantheine, based on the high degree of structural similarity to mitragynine, were also demonstrated.

### 2.3. Determination of the Alkaloid–CD Complex Stabilities by ACE

**Figure 3:**
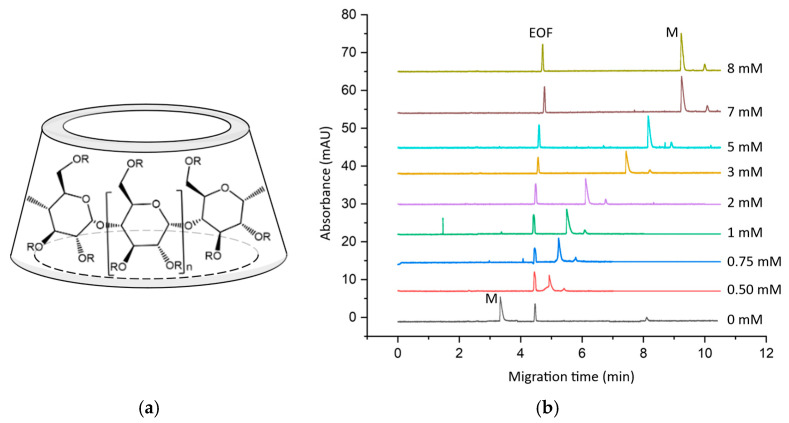
(**a**) Schematic structure of the cyclodextrins (R stands for substituents), (**b**) representative electropherograms of mitragynine (M)–sulfobutyl-ether-β-cyclodextrin DS~6.5 complexes in the presence of increasing cyclodextrin concentrations using dimethyl sulfoxide as the electroosmotic flow (EOF) marker. Different colours indicate different cyclodextrin concentrations. Additional conditions can be found in Section 3.7.

CD complexation can serve as an excellent tool to enhance the bioavailability of these kratom alkaloids. The large number of primary and secondary hydroxyl groups in the native CD molecule gives rise to various CD derivatives with a wide range of complexing abilities (see the schematic structure in Figure 3a). To investigate the CD complexation behavior of the four kratom alkaloids, we aimed to develop an ACE method for a systematic comparative study. The pH of the background electrolyte was chosen to be 7.4, reflecting physiological conditions, for which phosphate buffer was found to be appropriate. To ensure sufficient buffer capacity and to avoid excessive current and Joule heat generation, the buffer concentration was set to 30 mM, and the applied voltage was 15 kV. A representative set of electropherograms are shown in Figure 3b for several CD concentrations.

The most relevant, apparent, averaged complex stability constants are summarized in Table 2, while further details on the complex mobilities and additional stability values can be found in Table S5 in the Supplementary Materials.

The apparent, averaged complex stability constants show that complexation occurred with all three native CDs, but the highest stability values were obtained with the medium-cavity-sized β-CD for all four studied alkaloids (see Table 2). Comparing the differently substituted CD derivatives, the cavity size of β-CD was found to be optimal for complex formation (e.g., for the comparison of the carboxymethylated CD derivatives, see Table S5 in the Supplementary Materials).

Compared to native β-CD, its neutral derivatives formed complexes with similar stabilities; for the positively charged CD derivatives, no relevant stability constants could be observed due to the repulsion of the positive charges of the complexation agents and the analytes (see Table S5 in the Supplementary Materials).

The use of negatively charged sulfobutylated-β-CD was beneficial for complex formation compared to the native-β-CD in our previous study [13]. To reinforce and to extend this statement for the coalkaloids, a large number of negatively charged β-CD derivatives were tested, and, in most of the cases, the complexation affinity was increased for all four guest molecules. All carboxylated derivatives showed slightly increased stability compared to the native β-CD. Among the carboxylated derivatives, succinyl-β-CD (Succ-β-CD) showed the most significant complex formation ability due to the introduction of a polar OH group to the sidechain, which further increased the strength of the formed interactions. In the case of the phosphated β-CD derivative (Phos-β-CD), similar complex stabilities could be observed; however, the replacement of carboxyalkyl or phosphate groups with sulfate groups had a remarkable effect on complex formation. The complex stability constants increased with increasing alkyl chain length between the CD core and the negatively charged sulfate group and with the degree of substitution (DS) of the CD. For all four alkaloids, the complexes formed with SBE-β-CD DS~10.4 had the highest stability constants, almost reaching 10,000 M^−1^ for speciogynine.

In addition to the cavity size, the substituent type, and the degree of substitution of the CD, the position of the substituent on the CD rim also influenced the complex formation abilities of the studied alkaloids. Comparing the single isomer primary side persulfated HS-β-CD to its randomly sulfated analog, S-β-CD (DS~13), it is clearly shown that (in addition to the effect of the number of sulfate groups) substitution only on the primary side (thus the lack of negatively charged groups on the secondary side) impairs complex formation for the alkaloids. Additional single isomer persulfated CDs were investigated, with modifications on the secondary side: the 2,3-peracetylated (HDAS-β-CD), and the 2,3-permethylated (HDMS-β-CD) derivatives highlighting the positive effect of the polar acetyl group on the secondary side.

Additional special persubstituted thiopropionic acid CD derivatives, Sugammadex and its alpha (Sualfadex) and beta (Subetadex) analogs were included in this screening study. All Sugammadex analogs exhibited increased complex formation abilities compared to the native CDs, which were more pronounced in the case of Subetadex and Sugammadex, where the complex stability constants exceed 1000 M^−1^. Paynantheine and speciociliatine formed higher affinity complexes with the beta analog, while, in the case of mitragynine, the stability of the complex with the gamma analog Sugammadex (2050 M^−1^) even exceeded the affinity of the beta analog. Comparing Subetadex with its randomly substituted *O* analog (CE-β-CD), a significant increase in stability was achieved by substituting the primary side of the complexing agent and changing the heteroatom (*O* instead of *S*).

The results of our comprehensive, systematic ACE studies helped us to explore the main factors influencing complex formation and facilitated the selection of promising CD excipients for phase-solubility measurements.

In addition to conducting the ACE measurements for interaction studies, capillary electrophoresis is a widely used technique for the separation of stereoisomers or structurally similar compounds. Although there are several (U)HPLC, GC, or SFC methods for the qualitative or quantitative analysis of mitragynine and/or several kratom alkaloids [16], only one nonaqueous capillary electrophoretic method (NACE) with MS detection is available in the literature for the study of the mitragynine-type and paynantheine-type stereoisomers in kratom samples [28]. Our aim was to demonstrate the applicability of CD-based aqueous capillary electrophoresis systems capable of kratom alkaloid separations. Injecting samples containing the four alkaloids into the background electrolyte without CD, coelution of the alkaloid peaks occurred. Adding, e.g., CM-α-CD to the background electrolyte, separation could be observed at CD concentrations as low as 0.25 mM. Figure S15 shows the baseline separation of the four alkaloids with 1 mM CM-α-CD in pH 7.4 phosphate buffer, where *Rs* values were 3.2, 2.5, and 2.7.

### 2.4. Phase-Solubility Studies

Based on our ACE measurements, the promising CDs were selected for phase-solubility studies, along with the most widely used CD excipients, to investigate the effect of CD type and its concentration on mitragynine solubility. The experiments were conducted under conditions similar to those in the ACE measurements, applying 30 mM phosphate buffer (pH 7.4) and room temperature.

**Figure 4:**
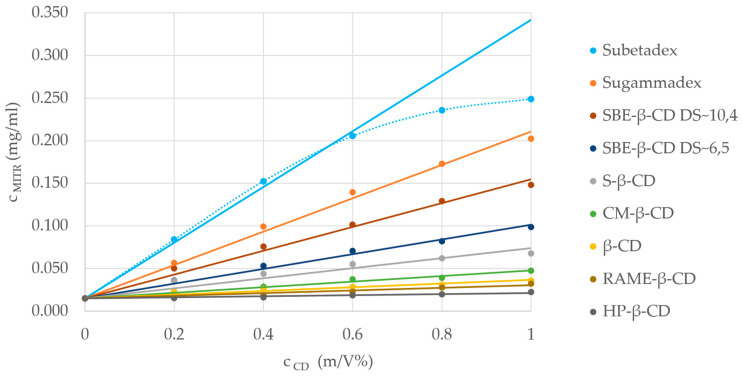
A_L_- and A_N_-type isotherms of mitragynine–cyclodextrin complexes. Additional conditions and cyclodextrin abbreviations can be found in Section 3.1 and Section 3.8. The R^2^ values for each fitting are listed in Table 3.

Most of the studied CDs showed an A_L_-type isotherm, representing a linear increase with constant stoichiometry (see Figure 4 for the phase-solubility isotherms). In the case of Subetadex, which had the highest solubility enhancement, an A_N_-type isotherm could be observed with a negative deviation from linearity, indicating a complex process: either the host ratio inside the complex increased, the solvent–solute interactions altered, or both contributed simultaneously.

The complex stability constants calculated from the phase-solubility study (Table 3) were in good correlation with the ACE results, reinforcing the higher complex formation ability of the studied alkaloids with negatively charged CD derivatives. However, in the ACE experiments, SBE-β-CD (DS~10.4) formed complexes with the highest stability constants; in the phase-solubility measurements, Subetadex and Sugammadex overperformed. Compared to the determination of complex stability constant, the determination of the complexation efficiency (CE) is a more accurate approach for evaluating the solubilizing efficiency of CDs, which may be crucial during the formulation development process. In the case of the Subetadex–mitragynine complex, the CE was 0.065 (calculated from the linear part of the isotherm); thus, 65 out of 1000 CD molecules formed a water-soluble complex with the poorly soluble mitragynine compared with the native β-CD, for which this value was only 2 out of 1000 (Table 3).

Our comparative CD complexation results demonstrate the ability of CDs to increase the solubility of kratom alkaloids. Our findings may simplify and drive further solubility and stability enhancement studies of these promising plant metabolites, enabling their therapeutic use.

## 3. Materials and Methods

### 3.1. Materials

The plant sample of *M. speciosa* leaves (green dragon) was obtained from Urban Garden Ltd. (Budapest, Hungary), which is of Indonesian origin, collected from the wild in West Sumatra. The average particle size of the kratom powder was 97 ± 22 μm, and the loss on drying was 6.30 ± 0.11%.

The preprocessed kratom extract used for the isolation of the alkaloids was purchased from Motark Enterprises (Hong Kong, Lot. No.: 19-09/041/X/45/B).

Dimethyl sulfoxide (DMSO), *n*-dodecane, hydrochloric acid (HCl), and sodium hydroxide (NaOH) were obtained from Reanal-Ker (Budapest, Hungary); methanol, dichloromethane, and gradient-grade acetonitrile were obtained from Molar Chemicals Ltd. (Halásztelek, Hungary). LC-MS-grade acetonitrile was from VWR chemicals (Radnor, PA, USA). Ammonium formate, caffeine, and rutin standards; phosphatidylcholine; cholesterol, porcine polar brain lipid extract; and the PBS (phosphate-buffered saline, pH 7.4) tablet were purchased from Merck (Darmstadt, Germany). Sodium dihydrogen phosphate (NaH_2_PO_4_), chloroform-*d*, and DMSO-*d*_6_ for NMR measurements were purchased from Sigma-Aldrich (Budapest, Hungary). All reagents were used without further purification. Bidistilled Millipore water was used throughout this study.

All native CDs (α, β and γ-CD) and their derivatives with various degrees of substitution were used in this study: dimethylated-β-CD DS~14 (DIME-β-CD), randomly methylated-β-CD DS~12 (RAME-β-CD), hydroxypropylated-β-CD DS~4.5 (HP-β-CD), hydroxypropylated-γ-CD DS~3 (HP-γ-CD), carboxymethylated-α-CD DS~3.5 (CM-α-CD), carboxymethylated-β-CD DS~3 (CM-β-CD), carboxymethylated-γ-CD DS~4 (CM-γ-CD), carboxyethylated-β-CD DS~3 (CE-β-CD), succinyl-β-CD DS~6 (Succ-β-CD), hexakis-(6-deoxy-6-(2-carboxyethyl)thio)-α-CD (Sualfadex), heptakis-(6-deoxy-6-(2-carboxyethyl)thio)-β-CD (Subetadex), octakis(6-deoxy-6-(2-carboxyethyl)thio)-γ-CD (Sugammadex), phosphated-β-CD DS~2-6 (Phos-β-CD), sulfobutyl-ether-β-CD DS~4, DS~6.5 and DS~10.4 (SBE-β-CD), sulfopropylated-β-CD DS~2 (SP-β-CD), sulfated-β-CD DS~13 (S-β-CD), sulfated-γ-CD DS~14 (S-γ-CD), heptakis-(6-*O*-sulfo)-β-CD (HS-β-CD), heptakis-(2,3-*O*-dimethyl, 6-*O*-sulfo)-β-CD (HDMS-β-CD), heptakis-(2,3-*O*-diacethyl, 6-*O*-sulfo)-β-CD (HDAS-β-CD), heptakis-(2-*O*-methyl, 3,6-*O*-disulfo)-β-CD (HMDiSu-β-CD), hexakis-(2,3-*O*-dimethyl, 6-*O*-sulfo)-α-CD (HxDMS-α-CD), octakis-(2,3-*O*-dimethyl, 6-*O*-sulfo)-γ-CD (ODMS-γ-CD), mono-(6-*N*-amino-6-deoxy)-β-CD (MA-β-CD), mono-6^A^-(3-hydroxy)propylamino-β-CD (HPA-β-CD), 6-monodeoxy-6-pyrrolidine-β-CD (PYR-β-CD), 6-monodeoxy-6-piperidine-β-CD (PIP-β-CD), mono-6^A^-(*N*-methyl-pyrrolidine)-β-CD (MePYR-β-CD), mono-6^A^-(*N*-methyl-piperidine)-β-CD (MePIP-β-CD), which were products of CycloLab Ltd. (Budapest, Hungary).

### 3.2. Extraction Procedures

#### 3.2.1. Laboratory-Scale Extraction Methods

Conventional extractions with 96% ethanol, *n*-pentane, and methanol in a laboratory Soxhlet extraction apparatus were carried out on raw, ground plant materials. Maceration was carried out in a stirred round-bottom flask (250 mL) at 40 and 50 °C for 10, 30 min, and 3 h using methanol and methanol acidified with HCl and different feed-to-solvent ratios (g/mL). Ultrasound-assisted extraction (UAE) was carried out using an ultrasound processor (Hielscher UP200St, Teltow, Germany) at a working frequency of 26 kHz, set at the highest amplitude (100%), with continuous pulse (100%) and power between 33 and 86 W (depending on the raw material) for 10 min at a temperature between 40 and 50 °C. Different solvents (ethanol and methanol) and different raw material-to-solvent ratios were applied.

After all extractions, the solvent was separated from the residue by filtration, and the filtrate was evaporated by a vacuum evaporator or with a TurboVap Automated Evaporator (TurboVap II, Biotage, Sweden). The extracts were weighed and collected into sample bottles. The solvent-free extracts were stored under nitrogen in a refrigerator until further analysis.

#### 3.2.2. Pilot-Scale Supercritical Fluid Extraction

The high-pressure extractions using CO_2_ solvent in the supercritical state were performed in a pilot plant apparatus with one 5 L extraction vessel (supplied by Natex Prozesstechnologie GesmbH, Ternitz, Austria) and two 1 L separation vessels in series. The carbon dioxide was added with a high-pressure liquid pump (LEWA EH-1, Lewa Herbert Ott GmbH, Leonberg, Germany), while cosolvent was added by another liquid pump (geared pump A 0.63/16R, TRIK-Pumpen GMBH, Kiel, Germany). Detailed operation details of the apparatus are described in a paper by Vagi et al. [29]. The high-pressure extraction was conducted at 30 MPa and 40 °C using supercritical CO_2_ + 10% (*v*/*v*), EtOH as a co-solvent, and the CO_2_ mass flow maintained at 7 kg/h.

### 3.3. UHPLC-UV Method

For the quantitative analysis of the variously treated kratom extracts, samples acquired in the PAMPA-BBB experiments and samples of the phase-solubility studies were analyzed with a Waters Acquity UHPLC system equipped with a diode array detector (Waters Corporation, Milford, MA, USA) using an Acquity BEH C18 column (2.1 × 100 mm; 1.7 µm, 45 °C) (Waters Corporation) and 2 mM ammonium formate at pH 10 as eluent A and acetonitrile as eluent B with a flow rate of 0.4 mL/min (from 80:20 to 0:100 in 9 min). UV spectra and chromatograms were recorded at 200–400 nm, and the chromatogram obtained at 226 nm was used for quantitative analyses.

The UHPLC-UV method was validated in accordance with the corresponding ICH guideline [17] in terms of specificity, selectivity, precision, linearity, LOD, LOQ, and accuracy. Blank samples (pure solvents, buffers) were analyzed to exclude any coelution of impurities with the analytes. Chromatograms were checked for sufficient separation of all 4 kratom alkaloids. Linearity was determined by analyzing mitragynine, speciogynine, speciociliatine, and paynantheine solutions at seven concentrations (0.5, 1, 5, 10, 50, 100, 130 μM in DMSO), each in triplicate. The slope, intercept, and correlation coefficient were determined by a least-squares weighted regression analysis. The LOD parameter was determined at a 3/1 and the LOQ at a 10/1 signal-to-noise ratio. Method precision was evaluated in terms of the relative standard deviation percentage (RSD%) of the retention times of the 4 examined alkaloids. The retention time’s repeatability was checked with six successive runs of the samples at three concentration levels (0.5 μM, 10 μM, 130 μM). To determine the accuracy of the method, recovery measurements were performed at three concentration levels (0.5 μM, 10 μM, 130 μM) for all the studied compounds. The experiments were conducted three times for each concentration, and the average percentage accuracy was determined for each compound.

### 3.4. Parallel Artificial Membrane Permeability Assay

PAMPA-BBB was used to determine the effective permeability (Pe) for some extracts and the isolated compounds. Stock solutions (10 mM in DMSO) were diluted with the buffer (pH 7.4) to obtain the donor solutions (composition: 594.0 μL buffer + 6.0 μL stock solution). The buffer was prepared as follows: one PBS (phosphate-buffered saline, pH 7.4; Sigma Aldrich, Budapest, Hungary) tablet dissolved in 200.0 mL distilled water. Donor solutions were filtered through Phenex-RC 15 mm, 0.2 μm syringe filters (Gen-Lab Ltd., Budapest, Hungary).

For PAMPA-BBB, 5 μL of porcine polar brain lipid extract (PBLE) solution (16.0 mg of PBLE + 8.0 mg of cholesterol dissolved in 600.0 μL of *n*-dodecane) was applied to each well of 96-well polycarbonate-based filter donor plates (top plate) (Multiscreen™-IP, MAIPN4510, pore size 0.45 μm; Merck). The 96-well PTFE acceptor plates (bottom plates) (Multiscreen Acceptor Plate, MSSACCEPTOR; Merck) were filled with 300.0 μL of buffer solution (0.01 M PBS buffer, pH 7.4). The donor plate was placed upon the acceptor plate, and both plates were incubated together at 37 °C for 4 h in a Heidolph Titramax 1000 Vibrating platform shaker (Heidolph, Schwabach, Germany).

After incubation, sandwich plates were separated, and the concentrations of each compound in the starting donor solution and in the acceptor and donor wells were determined in triplicate by the chromatographic peak areas derived from the UHPLC-UV method described above. The UV spectra and chromatograms were recorded at 200–400 nm, and the chromatograms acquired at the UV absorption maxima of each compound were used for data evaluation. The effective permeability and the membrane retention in PAMPA-BBB were calculated by Equations (1) and (2), respectively [30]:

(1) $$P_{e}=\frac{-2.303}{A(t-\tau_{SS})}\cdot (\frac{V_{A}\cdot V_{D}}{V_{A}+V_{D}})\cdot lg[1-(\frac{V_{A}+V_{D}}{(1-MR)\cdot V_{D}})\times (\frac{C_{A}(t)}{C_{D}(0)})]$$

where *P_e_* is the effective permeability coefficient (cm/s), *A* is the filter area (0.24 cm^2^), *V_D_* and *V_A_* are the volumes in the donor (0.15 cm^3^) and acceptor phases (0.30 cm^3^), *t* is the incubation time (s), *τ_SS_* is the time (s) to reach the steady state (240 s), *C_D_*(*t*) is the concentration (mol/cm^3^) of the compound in the donor phase at time *t*, *C_D_*(0) is the concentration (mol/cm^3^) of the compound in the donor phase at time 0, and MR is the estimated membrane retention factor (the estimated mole fraction of solute lost to the membrane), defined as

(2) $$MR=1-\frac{C_{D}(t)}{C_{D}(0)}-\frac{V_{A}}{V_{D}}\frac{C_{A}(t)}{C_{D}(0)}$$

All experiments were performed in triplicate on three consecutive days (n = 9); caffeine standard was used as the positive control, with rutin as the negative control.

### 3.5. Isolation Procedure

The preprocessed kratom extract (6.0 g) was separated using pilot-scale centrifugal partition chromatography (ECOM ECB2005PC Gradient Box with PC, ECOM ECP 2300 Isocratic Pump, ECOM Flash 14 DAD 400 UV-detector, ECOM Box with 10-position valve fraction collector, RotaChrom rCPC rotor with 2.1 L total column volume, and 100 extraction cells, Rotachrom Technologies LLC, Kecskemét, Hungary) procedure with a solvent system of dichloromethane:methanol:30 mM HCl in water (8:3:4 *v*/*v*/*v*) in descending mode with a flow rate of 150 mL/min. The injected sample (300 mL) was dissolved in the lower, organic phase, and elution–extrusion, ballast removal with pH control was applied before. The rCPC separation yielded eight fractions (*a*–*h*). Figure S16 demonstrates the alkaloid-rich fractions of the rCPC separation.

Fractions *c* (393.6 mg) and *e* (1037.8 mg) were further fractionated by reversed-phase flash chromatography (CombiFlash Nextgen 100, Teledyne Isco, Lincoln, NE, USA) on a RediSep Rf Gold^®^ 100 g reversed-phase C18 column (Teledyne Isco, USA) using 2 mM ammonium formate at pH 10 as eluent A and acetonitrile as eluent B, with a flow rate of 50 mL/min (0–3 min: 80:20; 3–12 min: 80:20 to 40:60; 12–25 min: 40:60 to 0:100; 25–30 min: 0:100). UV detection was performed at 200–400 nm. Fraction *c* yielded nine subfractions (*c*1–9), while fraction *e* yielded seven subfractions (*e*1–7). Fraction *c*6 produced speciociliatine (126.3 mg, >99% HPLC-UV), fraction *e*4 provided paynantheine (92.5 mg, >95% HPLC-UV), and fraction *e*6 yielded mitragynine (150.2 mg, >95% HPLC-UV). Fraction *e*5 (42.1 mg) was purified by semipreparative HPLC using a Kinetex EVO C18 column (150 × 10 mm, 5 μm, 45 °C, Phenomenex Inc. Torrance, CA, USA) and 2 mM ammonium formate at pH 10 as eluent A and acetonitrile as eluent B (from 50:50 to 30:70 *v*/*v* in 40 min) with a flow rate of 2.5 mL/min to yield speciogynine (2.5 mg, >95% HPLC-UV). The detection wavelength was set to 226 nm.

### 3.6. NMR

NMR experiments were carried out on a 600 MHz Varian DDR NMR spectrometer equipped with a 5 mm inverse-detection gradient (IDPFG) probehead at 298 (mitragynine, paynantheine), 333 (speciociliatine), and 343 K (speciogynine). Standard pulse sequences and processing routines available in VnmrJ 3.2C/Chempack 5.1 and MestreNova 15.0.1. were used for structure identifications. The complete resonance assignments were established from direct ^1^H–^13^C, long-range ^1^H–^13^C, and scalar spin–spin connectivities using 1D ^1^H, ^13^C and 2D ^1^H–^1^H gCOSY, ^1^H–^1^H zTOCSY, ^1^H–^1^H NOESY, ^1^H–^13^C gHSQCAD (*J* = 140 Hz), and ^1^H–^13^C gHMBCAD experiments, respectively. Also, selective 1D zTOCSY experiments were carried out. NMR spectra were recorded in chloroform-*d* (mitragynine) and DMSO-*d6* (speciociliatine), in some cases acidified with TFA (paynantheine and speciogynine). The ^1^H and ^13^C chemical shifts were referenced to the applied NMR solvent (DMSO-*d6* and chloroform-*d*).

### 3.7. Affinity Capillary Electrophoresis

The ACE experiments were performed by using Agilent 7100 and HP^3D^CE instruments (Agilent Technologies, Waldbronn, Germany). Untreated fused silica capillaries (total length of 48.5 cm, effective length of 40 cm, inner diameter of 50 μm) were purchased from Agilent Technologies. The new capillaries were conditioned with a 1 M NaOH wash for 1 h, followed by a 30 min wash each with 0.1 M NaOH and purified water. Prior to all runs, the capillary was preconditioned by rinsing with background electrolyte for 3 min. During the measurements, 30 mM phosphate buffer at pH 7.4 (30 mM Na_2_HPO_4_-NaOH) was used. The background electrolyte contained CDs at various concentrations. Stock solutions of the studied kratom alkaloids (mitragynine, paynantheine, speciogynine, speciociliatine) were prepared separately in methanol (1 mg/mL), and, right before the measurements, appropriate dilutions with purified water were used to prepare the sample solutions, applying 0.1% DMSO as the electroosmotic flow (EOF) marker. The samples were injected hydrodynamically at 40 mbar for 3 s. The positive polarity voltage was set to 15 kV, and the capillary was set to 25 °C. The electropherograms were recorded at four wavelengths (200 nm, 210 nm, 215 nm, and 220 nm), and the data were evaluated at 215 nm. In each case, three parallel measurements were performed. The measurements were evaluated using Origin and CEval software (v. 0.6i3) [31].

In order to determine the complex stability constants that characterized the strength of the complexes, the first step was to determine the effective mobility of the analyte at specific *CD* concentrations. Triangular peaks caused by electromigration dispersion are often observed in ACE measurements. To suppress these errors, *HVL* Function (3) can be used to determine the effective mobility more accurately:

(3) $${HVL}_{\delta }\left(t;a_{0},a_{1},a_{2},a_{3\delta }\right)=\frac{\frac{a_{0}}{a_{2}a_{3\delta }\sqrt{2\pi }}exp\left[{-\frac{1}{2}\left(\frac{t-a_{1}}{a_{2}}\right)}^{2}\right]}{\frac{1}{exp\left(a_{3\delta }\right)-1}+\frac{1}{2}\left[1+erf\left(\frac{t-a_{1}}{\sqrt{2a_{2}}}\right)\right]}\;$$

where *a*_0_ is the area of the HVL function, *a*_1_ is the position of the Gaussian component corresponding to the migration time of the analyte, *a*_2_ is the standard deviation of the Gaussian component, and *a*_3_*_δ_* is the triangular distortion.

Assuming a 1:1 complexation ratio and that the equilibrium *CD* concentration is almost equal to the total *CD* concentration, a relationship between the effective mobility relative to the EOF and the complex stability constant can be described by Function (4)

(4) $$\mu_{eff}=\frac{\mu_{A}+\mu_{ACD}K_{stab}\left[CD\right]}{1+K_{stab}\left[CD\right]}$$

where *µ_eff_* is the effective mobility of the analyte, *µ_A_* and *µ_ACD_* are the free and complexed analyte mobilities, [*CD*] is the concentration of the selector, and *K_stab_* is the complex stability constant.

In order to calculate the exact, apparent, averaged complex stability constant values, viscosity correction [32] and ionic strength correction [33] were used.

### 3.8. Phase-Solubility Studies

Solubility measurements were performed according to Higuchi and Connors [34]. To each 1 mg mitragynine sample, the selected cyclodextrins were added. The final concentration of the CDs was in the range of 0–1 (*m*/v) %. Suspensions were shaken at room temperature for 24 h and filtered using 0.22 μm filters. The mitragynine concentration in the samples was determined by the above-described UHPLC-UV method. The solubility diagrams were constructed, and the stability constant (*K_stab_*) and complexation efficacy (CE) were calculated using the following formulas:

(5) $$K_{stab}=\frac{slope}{S_{0}(1-slope)}$$

(6) $$CE=\frac{slope}{1-slope}$$

where *S*_0_ is the intrinsic solubility of a poorly soluble drug. The experiments were performed in a 30 mM phosphate buffer (pH 7.4).

## 4. Conclusions

The characteristic alkaloid component of *M. speciosa* leaves, mitragynine, is a promising secondary plant metabolite with potential future therapeutic applications in the treatment of opioid withdrawal symptoms and analgesia. However, considering the long-standing medicinal use of complex kratom extracts, the contribution of the coalkaloids of mitragynine to the promising therapeutic effects cannot be neglected. Given that these effects are primarily related to CNS activity, BBB penetration can be considered critical for all active constituents in kratom. To identify the key secondary metabolites in kratom extracts that can cross the BBB, various extraction methods were tested, which was followed by a PAMPA-BBB study. Four main BBB+ alkaloids were isolated and identified as mitragynine, paynantheine, speciociliatine, and speciogynine. The CD complexation ability of the four kratom alkaloids was screened via affinity capillary electrophoresis, and the promising CDs were selected for phase-solubility studies along with the most widely used CD excipients to increase the bioavailability of mitragynine. The medium-cavity-size β-CD derivatives proved to be the most promising complexing agents. By examining the charge, the type, and the number of the substituents, the presence of negatively charged substituents (e.g., in the case of the sulfoalkylated SBE-β-CD) with increasing DSs led to a significant increase in complex formation abilities. Furthermore, special CD derivatives, the persubstituted thiopropionic acid CD derivatives, Sugammadex and Subetadex, can significantly increase the alkaloid–CD complex stabilities and the solubility of mitragynine. The results of our complex formation study can facilitate bioavailability enhancement; and the design of further drug formulation studies, furthermore, may lead to the identification of CD-based antidotes for kratom alkaloids.

## Figures and Tables

**Table 1:** Results of PAMPA experiments expressed as log*P_e_* values (*n* = 9). Further conditions can be found in Section 3.4.

Compound	log*P_e_* PAMPA-BBB (*n* = 9)
Mitragynine	−4.42 ± 0.61
Paynantheine	−4.61 ± 0.31
Speciociliatine	−4.58 ± 0.85
Speciogynine	−4.77 ± 0.42

**Table 2:** Alkaloid–cyclodextrin complex stability constants (M^−1^) measured via affinity capillary electrophoresis with 30 mM phosphate buffer (pH 7.4), 25 °C, 15 kV, 215 nm. In some cases, cyclodextrins with different degrees of substitution (DSs) were used for the complexation study. Further conditions and cyclodextrin abbreviations can be found in Section 3.1 and Section 3.7.

	Cyclodextrin	Mitragynine	Paynantheine	Speciociliatine	Speciogynine
**Native CDs**	**α-CD**	25 ± 2	20 ± 2	22 ± 3	23 ± 5
	**β-CD**	145 ± 20	145 ± 15	35 ± 3	65 ± 9
	**γ-CD**	55 ± 5	140 ± 5	15 ± 1	45 ± 4
**Negatively** **charged CD** **derivatives**	**Succ-β-CD**	375± 20	360 ± 30	470 ± 35	840 ± 80
	**Phos-β-CD**	290 ± 30	335 ± 30	215 ± 15	420 ± 30
	**SBE-β-CD** **DS~4**	550 ± 35	830 ± 50	670 ± 60	610 ± 60
	**SBE-β-CD** **DS~6.5**	1530 ± 55	2000 ± 75	1700 ± 75	1800 ± 100
	**SBE-β-CD** **DS~10.4**	4900 ± 420	5300 ± 280	5530 ± 435	9200 ± 890
	**S-β-CD**	1650 ± 30	1420 ± 145	2410 ± 185	970 ± 80
	**HS-β-CD**	1250 ± 105	575 ± 35	850 ± 30	645 ± 30
	**HDAS-β-CD**	4100 ± 180	1720 ± 125	3000 ± 275	2350 ± 115
	**HDMS-β-CD**	175 ± 7	135 ± 14	105 ± 12	30 ± 3
	**Subetadex**	1800 ± 80	3600 ± 300	2700 ± 100	n.d.
	**Sugammadex**	2050 ± 125	2750 ± 175	700 ± 45	n.d.

n.d.: not determined.

**Table 3:** Mitragynine–cyclodextrin (CD) complex stability constants (K_stab_, M^−1^) and complexation efficacy (CE) measured by high-performance liquid chromatography, with the R^2^ values of the linear fitting listed in Figure 4. In some cases, CDs with different degrees of substitution (DSs) were used for the complexation study. Additional conditions and CD abbreviations can be found in Section 3.1, Section 3.3 and Section 3.8.

Cyclodextrin		K_stab_	CE	R^2^
**Native CD**	**β** **-CD**	55	0.0021	0.9463
**Neutral CD** **derivatives**	**RAME-β-CD**	60	0.0023	0.9598
	**HP-β-CD**	30	0.0011	0.9334
**Negatively charged CD** **derivatives**	**CM-β-CD**	300	0.0113	0.9849
	**Subetadex**	1730	0.0650	0.9964
	**Sugammadex**	1150	0.0431	0.9953
	**SBE-β-CD DS~6.5**	480	0.0181	0.9933
	**SBE-β-CD DS~10.4**	1020	0.0382	0.9938
	**S-β-CD**	340	0.0127	0.9531

## Data Availability

Data are contained within this article.

## Supplementary Materials

The following supporting information can be downloaded at: https://www.mdpi.com/article/10.3390/molecules29225302/s1. Table S1: UHPLC-UV method validation and system suitability tests: linearity, limit of detection (LOD), limit of quantitation (LOQ) values, precision, and accuracy. Method precision was evaluated in terms of the relative standard deviation percentage (RSD%) of the retention times. Additional conditions can be found in Section 3.3. Table S2. Comparison of the applied extraction procedures (Soxhlet extraction, maceration, ultrasound-assisted extraction (UAE), and supercritical CO_2_ extraction (SFE)) regarding the yield and the mitragynine content. Additional conditions can be found in Section 3.2. Table S3. The mitragynine and coalkaloid contents (mg/g) using ultrasound-assisted extraction (UEA) with ethanol and methanol solvents at different temperatures. The UEA lasted 10 min, and the ratio of feed-to -solvent was 1:10 g/L in all cases. Table S4. Characteristic ^1^H and ^13^C NMR chemical shifts (in ppm) of the compounds. Additional conditions can be found in Section 3.6. Figure S1. ^1^H NMR spectrum of mitragynine (in CDCl_3_, at 298 K). Figure S2. ^13^C NMR spectrum of mitragynine in CDCl_3_. Figure S3. HSQC spectrum of mitragynine in CDCl_3_. Figure S4. ^1^H NMR spectrum of speciociliatine in DMSO-*d*_6_. Figure S5. ^13^C NMR spectrum of speciociliatine in DMSO-*d*_6_. Figure S6. HSQC spectrum of speciociliatine in DMSO-*d*_6_. Figure S7. NOESY spectrum of speciociliatine in DMSO-*d*_6_. Figure S8. ^1^H NMR spectrum of speciogynine in DMSO-*d*_6_. Figure S9. ^13^C NMR spectrum of speciogynine in DMSO-*d*_6_. Figure S10. HSQC spectrum of speciogynine in DMSO-*d*_6_. Figure S11. NOESY spectrum of speciogynine in DMSO-*d*_6_. Figure S12. ^1^H NMR spectrum of paynantheine in DMSO-*d*_6_. Figure S13. ^13^C NMR spectrum of paynantheine in DMSO-*d*_6_. Figure S14. HSQC spectrum of paynantheine in DMSO-*d*_6_. Table S5. Alkaloid–CD complex stability constants (M^−1^) measured by affinity capillary electrophoresis in 30 mM phosphate buffer (pH 7.4), 25 °C, 15 kV, 215 nm. Additional conditions and CD abbreviations can be found in Section 3.1 and Section 3.7. Figure S15: Capillary electrophoretic separation of the four studied alkaloids (SC: speciociliatine, M: mitragynine, SG: speciogynine, P: paynantheine) using 1 mM carboxymethylated-α-cyclodextrin in 30 mM phosphate buffer (pH 7.4), 25 °C, 15 kV, 215 nm. Figure S16. Pilot-scale CPC chromatogram of the preprocessed kratom extract. Additional conditions can be found in Section 3.5.
