# Supplementary material for: Kratom Alkaloids: A Blood–Brain Barrier Specific Membrane Permeability Assay-Guided Isolation and Cyclodextrin Complexation Study
Source: Molecules. 2024 Nov 9;29(22):5302. doi: 10.3390/molecules29225302 (PMC11597089; doi:10.3390/molecules29225302)
Supplement: Supplementary file 1 [file molecules-29-05302-s001.zip › molecules-3231203-supplementary.pdf]

Supplementary Material for Article

Kratom Alkaloids: A Blood–Brain Barrier Specific Membrane Permeability Assay-Guided Isolation and Cyclodextrin Complexation Study

András Dohárszky [1,2], Erika Mária Vági [3], Árpád Könczöl [4], Alexandra Simon [1,2], Erzsébet Várnagy [1,2,3], Miras Muratov [3], Kristóf István Steiger [1], Bianka Várnai [1], Szabolcs Béni [5,1], Eszter Riethmüller [1,2]*, and Ida Fejős [1,2]*

1 Department of Pharmacognosy, Semmelweis University, Üllői út 26, H-1085 Budapest, Hungary; doharszky.andras@stud.semmelweis.hu; simon.alexandra1@semmelweis.hu; varnagy.erzsebet@phd.semmelweis.hu; stofi1998@gmail.com; varnai.bianka@phd.semmelweis.hu; riethmuller.eszter@semmelweis.hu; fejos.ida@semmelweis.hu

2 Center for Pharmacology and Drug Research & Development, Semmelweis University, Budapest, Hungary

3 Department of Chemical and Environmental Process Engineering, Faculty of Chemical Technology and Biotechnology, Budapest University of Technology and Economics, Műegyetem rkp. 3, H-1111 Budapest, Hungary; vagierikamaria@gmail.com; mirasmuratov1998@gmail.com

4 RotaChrom Technologies LLC, H-6000 Kecskemét, Hungary; konczol.capice@gmail.com

5 Integrative Health and Environmental Analysis Research Laboratory, Department of Analytical Chemistry, Institute of Chemistry, ELTE Eötvös Loránd University, Pázmány Péter sétány 1/A, H-1117 Budapest, Hungary; szabolcs.beni@ttk.elte.hu_

* Correspondence: E.R.: riethmuller.eszter@semmelweis.hu; I.F.: fejos.ida@semmelweis.hu

Content: Table S1: UHPLC-UV method validation and system suitability tests: linearity, limit of detection (LOD), limit of quantitation (LOQ) values, precision and accuracy. Method precision was evaluated in terms of the relative standard deviation percentage (RSD%) of the retention times. Further conditions can be found in *3.3. UHPLC-UV method* section. **Table S2.** Comparison of the applied extraction procedures (Soxhlet extraction, maceration, ultrasound-assisted extraction (UAE) and supercritical CO_2 extraction (SFE)), regarding the yield and the mitragynine content. Further conditions can be found in *3.2. Extraction procedures* section. **Table S3.** The mitragynine and co-alkaloid content (mg/g) using ultrasound assisted extraction (UEA) with ethanol and methanol solvents at different temperatures. The UEA lasted 10 minutes and the ratio of feed to solvent was 1:10 g/L in all cases. **Table S4.** Characteristic ^{1}H and ^{13}C NMR chemical shifts (in ppm) of the compounds. Further conditions can be found in *3.6. NMR* section. **Figure S1.** ^{1}H NMR spectrum of mitragynine (in $CDCl_3$, at 298 K). **Figure S2.** ^{13}C NMR spectrum of mitragynine in $CDCl_3$. **Figure S3.** HSQC spectrum of mitragynine in $CDCl_3$. **Figure S4.** ^{1}H NMR spectrum of speciociliatine in DMSO-d_6. **Figure S5.** ^{13}C NMR spectrum of speciociliatine in DMSO-d_6. **Figure S6.** HSQC spectrum of speciociliatine in DMSO-d_6. **Figure S7.** NOESY spectrum of speciociliatine in DMSO-d_6. **Figure S8.** ^{1}H NMR spectrum of speciogynine in DMSO-d_6. **Figure S9.** ^{13}C NMR spectrum of speciogynine in DMSO-d_6. **Figure S10.** HSQC spectrum of speciogynine in DMSO-d_6. **Figure S11.** NOESY spectrum of speciogynine in DMSO-d_6. **Figure S12.** ^{1}H NMR spectrum of paynantheine in DMSO-d_6. **Figure S13.** ^{13}C NMR spectrum of paynantheine in DMSO-d_6. **Figure S14.** HSQC spectrum of paynantheine in DMSO-d_6. **Table S5.** Alkaloid-CD complex stability constants (M^{-1}) measured by affinity capillary electrophoresis at 30 mM phosphate buffer (pH 7.4), 25°C, 15 kV, 215 nm. Further conditions and CD abbreviations can be found in *3.7. Affinity Capillary Electrophoresis* and *3.1. Materials* section. **Figure S15:** Capillary electrophoretic separation of the four studied alkaloids (SC: speciociliatine, M: mitragynine, SG: speciogynine, P: paynantheine) using 1 mM carboxymethylated-α-cyclodextrin in 30 mM phosphate buffer (pH 7.4), 25°C, 15 kV, 215 nm. **Figure S16.** Pilot-scale CPC chromatogram of the preprocessed kratom extract Further conditions can be found in *3.5. Isolation procedure* section.

Table S1: UHPLC-UV method validation and system suitability tests: linearity, limit of detection (LOD), limit of quantitation (LOQ) values, precision and accuracy. Method precision was evaluated in terms of the relative standard deviation percentage (RSD%) of the retention times. Further conditions can be found in *3.3. UHPLC-UV method* section.

Compound	Regression Equation	Weight factor	R^2	Regression range (µM)	LOD (µM)	LOQ (µM)
mitragynine	y=99.94x+59.41	1/x	0.9990	0.5-130	0.04	0.11
speciogynine	y=90.09x+4.02	1/x	0.9994	0.5-130	0.16	0.48
speciociliatine	y=92.30x+8.83	1/x	0.9995	0.5-130	0.14	0.42
paynantheine	y=147.51x+25.26	1/x	0.9995	0.5-130	0.04	0.13

Nominal conc. (µM)	Precision (RSD%)		Accuracy (%)	
	Intraday	Interday	Intraday	Interday
**mitragynine**				
130	0.91	1.58	98.51	96.90
10	2.26	5.33	108.71	103.78
0.5	3.97	7.28	95.39	93.05
**speciogynine**				
130	0.55	0.72	98.03	97.73
10	0.66	3.53	104.43	102.31
0.5	1.27	8.87	108.71	103.73
**speciociliatine**				
130	0.36	1.16	97.68	97.84
10	0.66	4.93	108.96	104.46
0.5	2.76	9.75	100.64	92.56
**paynantheine**				
130	0.69	0.61	97.43	97.64
10	0.77	1.93	103.35	105.79
0.5	2.91	7.74	91.90	86.98

Table S2. Comparison of the applied extraction procedures (Soxhlet extraction, maceration, ultrasound-assisted extraction (UAE) and supercritical CO_2 extraction (SFE) applying methanol (MeOH) and ethanol (EtOH)), regarding the yield and the mitragynine content. Further conditions can be found in *3.2. Extraction procedures* section.

**Method**	**Solvent**	**T (°C)**	**Time (min)**	**Feed to solvent ratio (g:L)**	**Yield (g/100 g dry matter)**	**Mitragynine content (mg/g)**
**Soxhlet**	**EtOH**	~ 78	1260	1:10	37.7 ± 2.8	34.3 ± 2.2
	**MeOH**	~ 65	1260	1:10	42.5 ± 1.0	36.7 ± 1.3
**Maceration**	**MeOH**	40	10	1:10	31.9 ± 0.4	54.4 ± 3.4
	**MeOH**	50	10	1:10	33.8 ± 0.3	47.7 ± 1.6
	**MeOH pH 2**	50	10	1:10	36.5 ± 0.3	48.9 ± 1.5
	**MeOH**	50	30	1:10	34.8 ± 0.2	51.2 ± 4.2
	**MeOH**	50	180	1:10	34.6 ± 0.2	48.8 ± 3.2
**UAE**	**EtOH**	30	10	1:10	27.6 ± 0.2	62.1 ± 4.1
		45	10	1:10	27.9 ± 0.3	65.3 ± 6.9
		60	10	1:10	29.7 ± 0.4	60.7 ± 1.8
	**MeOH**	30	10	1:10	30.9 ± 0.3	52.6 ± 3.2
		40	10	1:10	33.1 ± 0.3	50.0 ± 3.2
		45	10	1:10	34.4 ± 0.4	47.7 ± 1.4
		50	10	1:10	34.1 ± 0.3	49.6 ± 1.4
		50	10	1:5	32.5 ± 0.3	50.5 ± 0.7
		50	10	1:15	32.8 ± 0.4	47.8 ± 2.7
		60	10	1:10	32.7 ± 0.3	46.3 ± 7.2
**SFE/1**	**$scCO_2$ + 10% EtOH**	40	30	*n.d.*	1.02	14.3 ± 0.4
**SFE/2**		40	60		0.98	26.7 ± 1.9
**SFE/3**		40	90		0.69	40.0 ± 5.1
**SFE/4**		40	135		0.50	52.4 ± 0.3
**SFE/5**		40	180		0.35	68.6 ± 6.3
**SFE/6**		40	225		0.36	68.0 ± 5.7
**SFE/7**		40	270		0.36	70.0 ± 7.2
**SFE/8**		40	315		0.27	79.2 ± 4.8
**SFE/9**		40	360		0.30	59.6 ± 9.9
**SFE/10**		40	405		0.20	94.3 ± 8.4
**SFE/11-13**		40	450		0.20	87.1 ± 7.9

n.d.: not determined.

Table S3. The mitragynine and co-alkaloid content (mg/g) using ultrasound assisted extraction (UEA) with ethanol and methanol solvents at different temperatures. The UEA lasted 10 minutes and the ratio of feed to solvent was 1:10 g/L in all cases.

Solvent	T (°C)	Yield (g/100 g dry matter)	Mitragynine content (mg/g)	Speciogynine content (mg/g)	Speciociliatine content (mg/g)	Paynantheine content (mg/g)
**EtOH**	30	27.6 ± 0.2	62.1 ± 4.1	9.4 ± 0.3	13.9 ± 0.5	11.7 ± 0.4
	45	27.9 ± 0.3	65.3 ± 6.9	9.6 ± 1.1	14.5 ± 1.6	12.0 ± 1.3
	60	29.7 ± 0.4	60.7 ± 1.8	8.9 ± 0.3	13.4 ± 0.5	11.1 ± 0.3
**MeOH**	30	30.9 ± 0.3	52.6 ± 3.2	7.5 ± 0.9	11.5 ± 1.0	9.5 ± 0.7
	45	34.4 ± 0.4	47.7 ± 1.4	6.9 ± 0.1	10.1 ± 0.3	8.5 ± 0.2
	60	32.7 ± 0.3	46.3 ± 7.2	6.4 ± 0.2	10.9 ± 0.1	9.0 ± 0.1

Table S4. Characteristic ^{1}H and ^{13}C NMR chemical shifts (in ppm) of the compounds. Further conditions can be found in *3.6. NMR* section.

	**mitragynine**[a]		**speciogynine**[b]		**speciociliatine**[b]		**paynantheine**[b]	
	**^{1}H**	**^{13}C**	**^{1}H**	**^{13}C**	**^{1}H**	**^{13}C**	**^{1}H**	**^{13}C**
**3**	3.15	61.7	4.69	60.1	4.00	54.2	4.74	59.9
**17**	7.43	160.6	7.54	160.8	7.46	159.8	7.52	161.2
**18**	0.86	13.0	0.85	9.9	0.83	12.2	5.53<br>5.05	117.7
**9-OMe**	3.87	55.5	3.84	55.0	3.80	54.9	3.83	55.2
**17-OMe**	3.72	61.7	3.88	61.5	3.79	61.1	3.73	61.8
**22-OMe**	3.70	51.5	3.66	50.7	3.60	50.8	3.63	51.1

[a] in chloroform-*d*
[b] in DMSO-*d6*

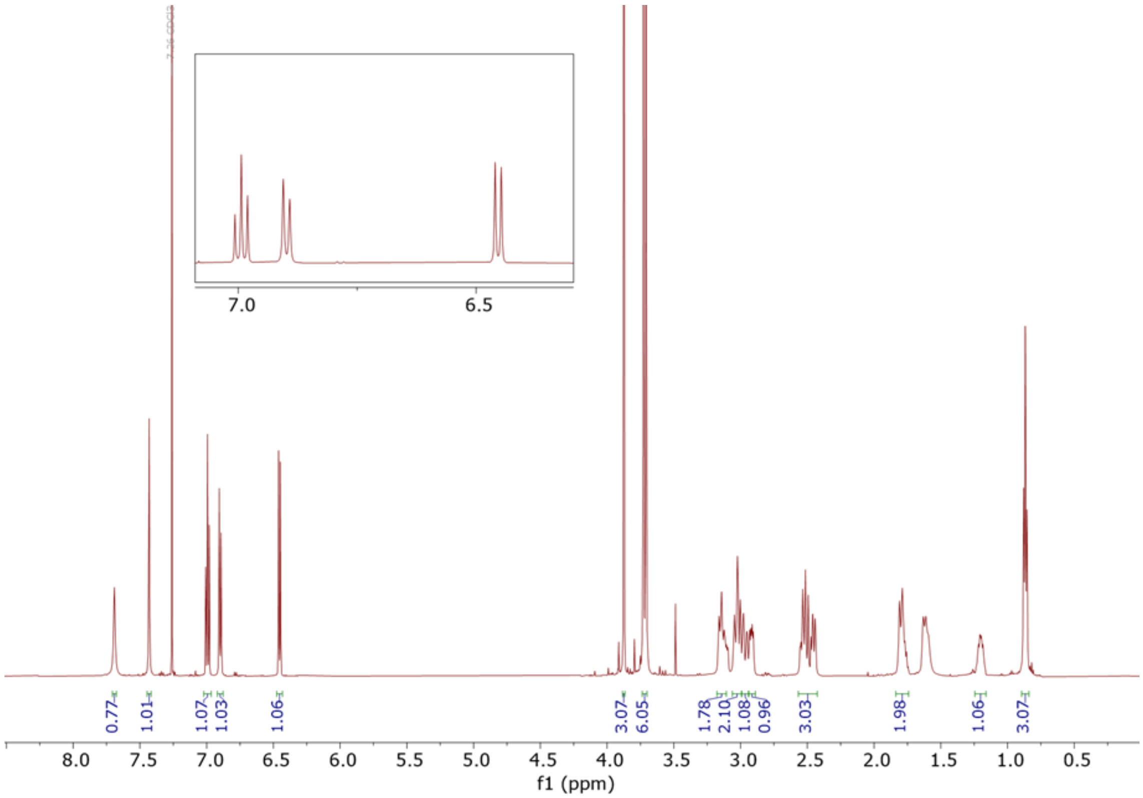

Figure S1. ^{1}H NMR spectrum of mitragynine (in $CDCl_3$, at 298 K).

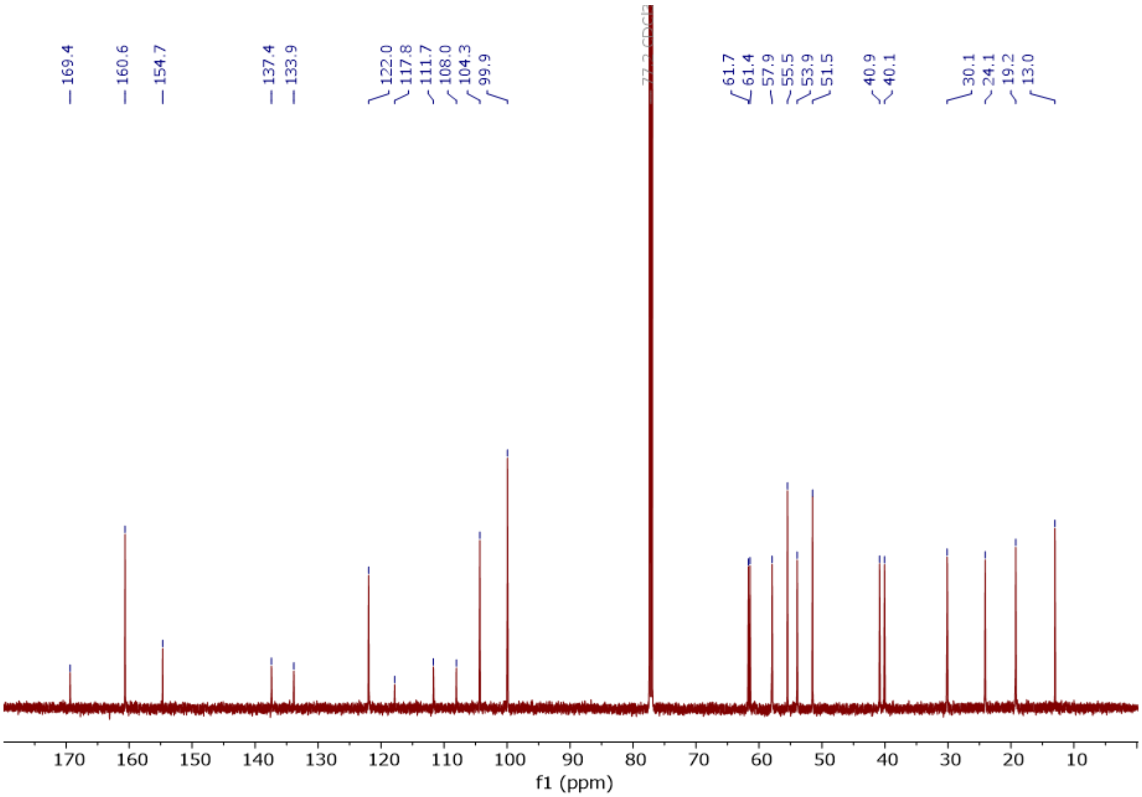

Figure S2. ^{13}C NMR spectrum of mitragynine in $CDCl_3$.

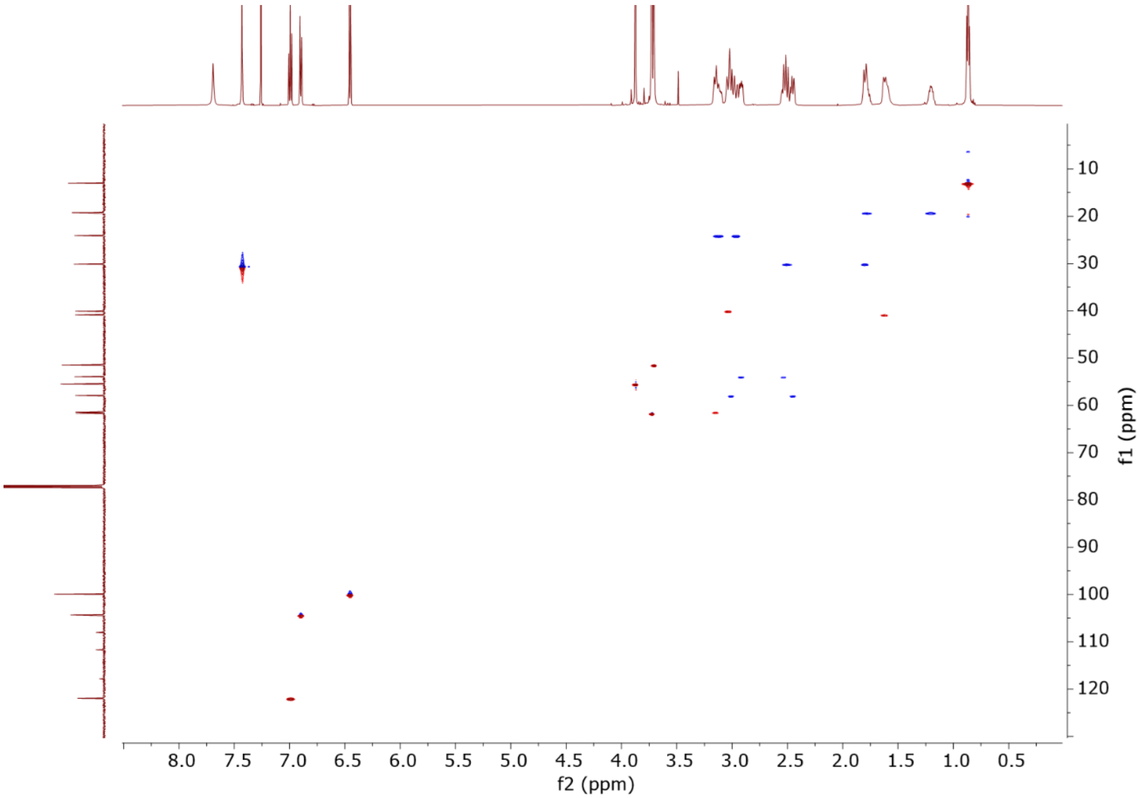

Figure S3. HSQC spectrum of mitragynine in $CDCl_3$.

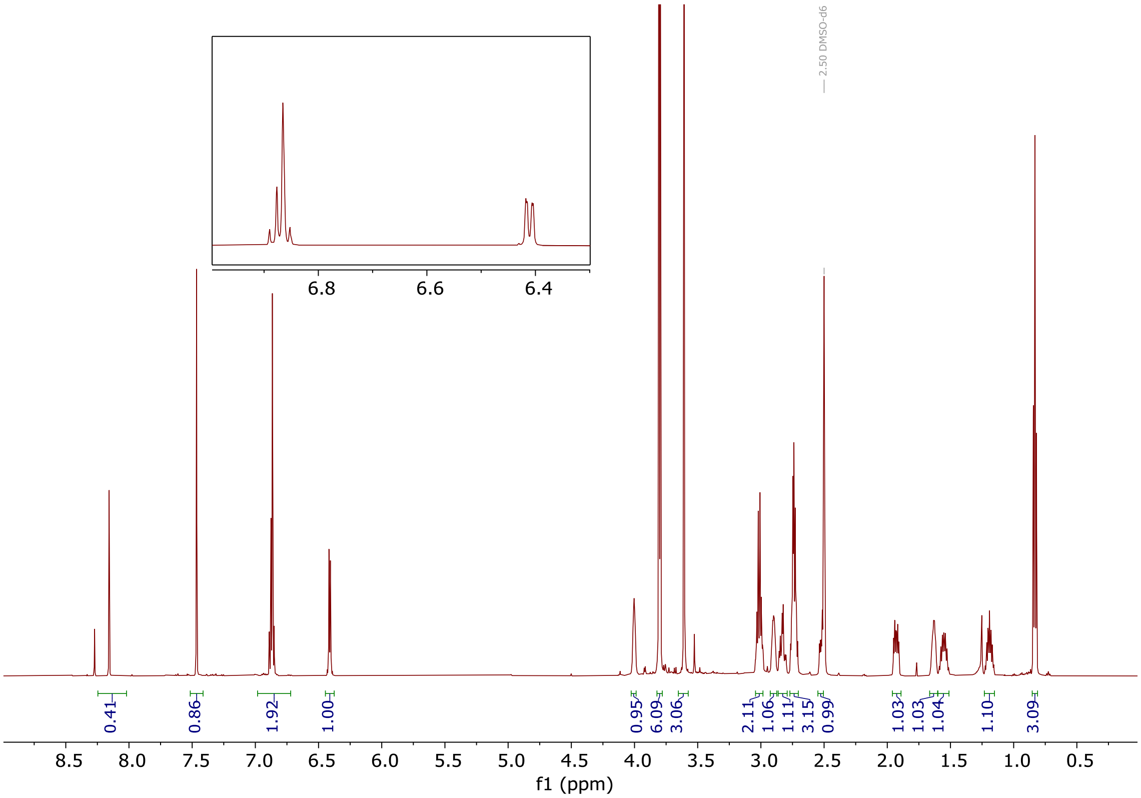

Figure S4. ^{1}H NMR spectrum of speciociliatine in DMSO-d_6.

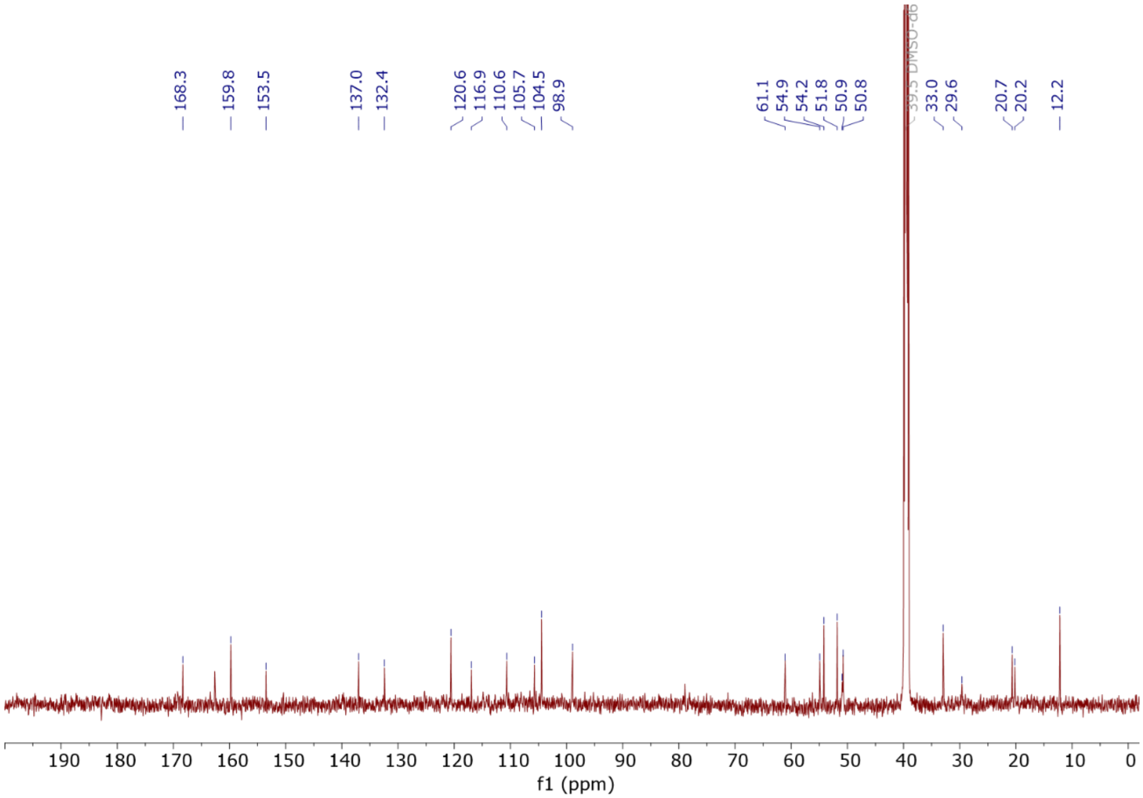

Figure S5. ^{13}C NMR spectrum of speciociliatine in DMSO-d_6.

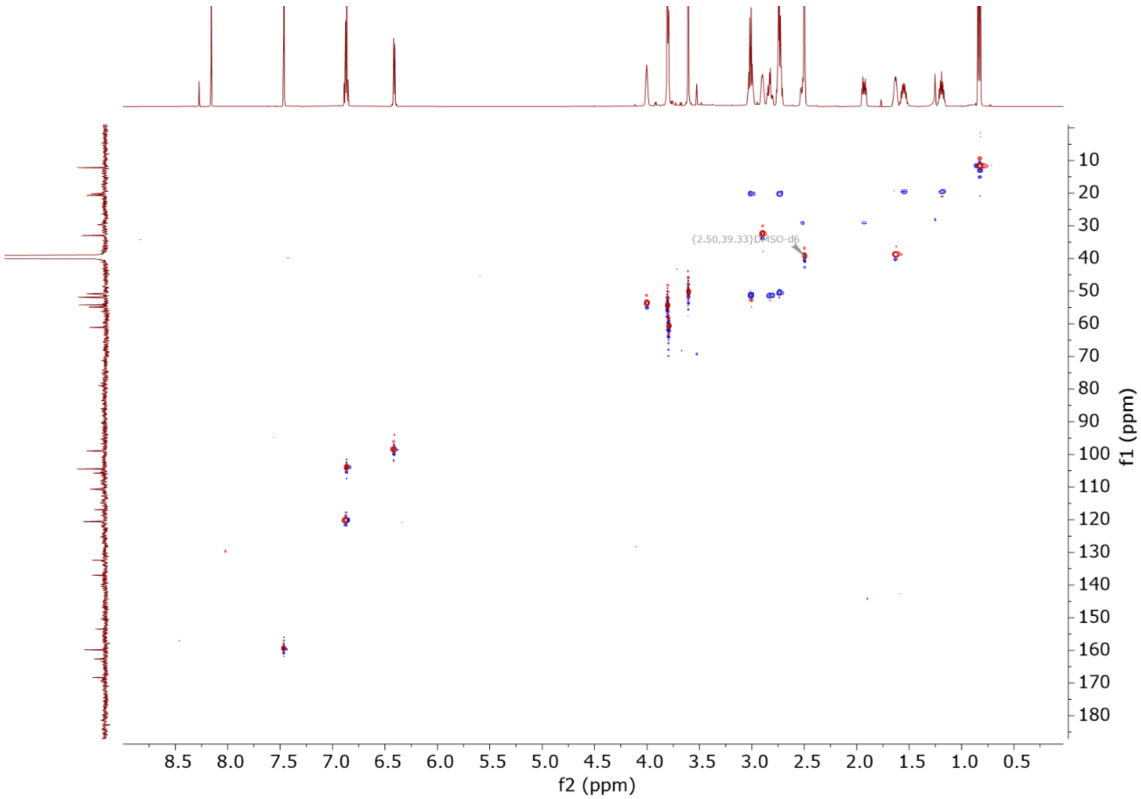

Figure S6. HSQC spectrum of speciociliatine in DMSO-d_6.

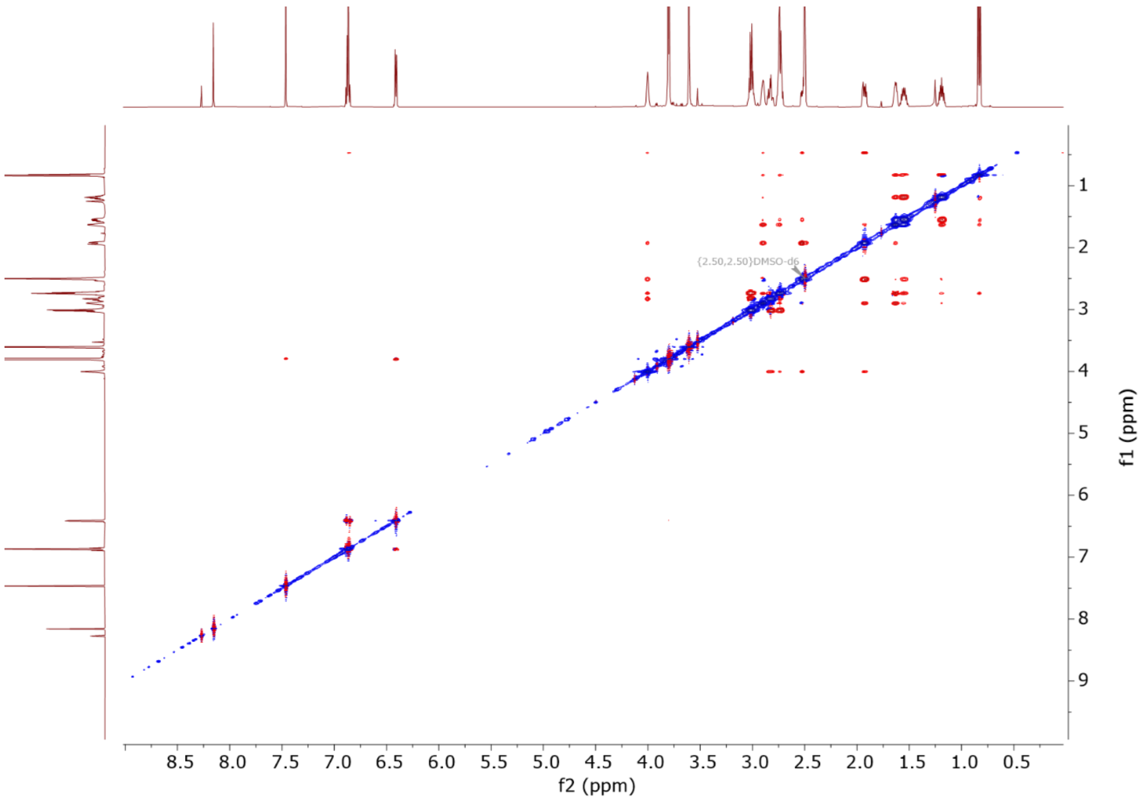

Figure S7. NOESY spectrum of speciociliatine in DMSO-d_6.

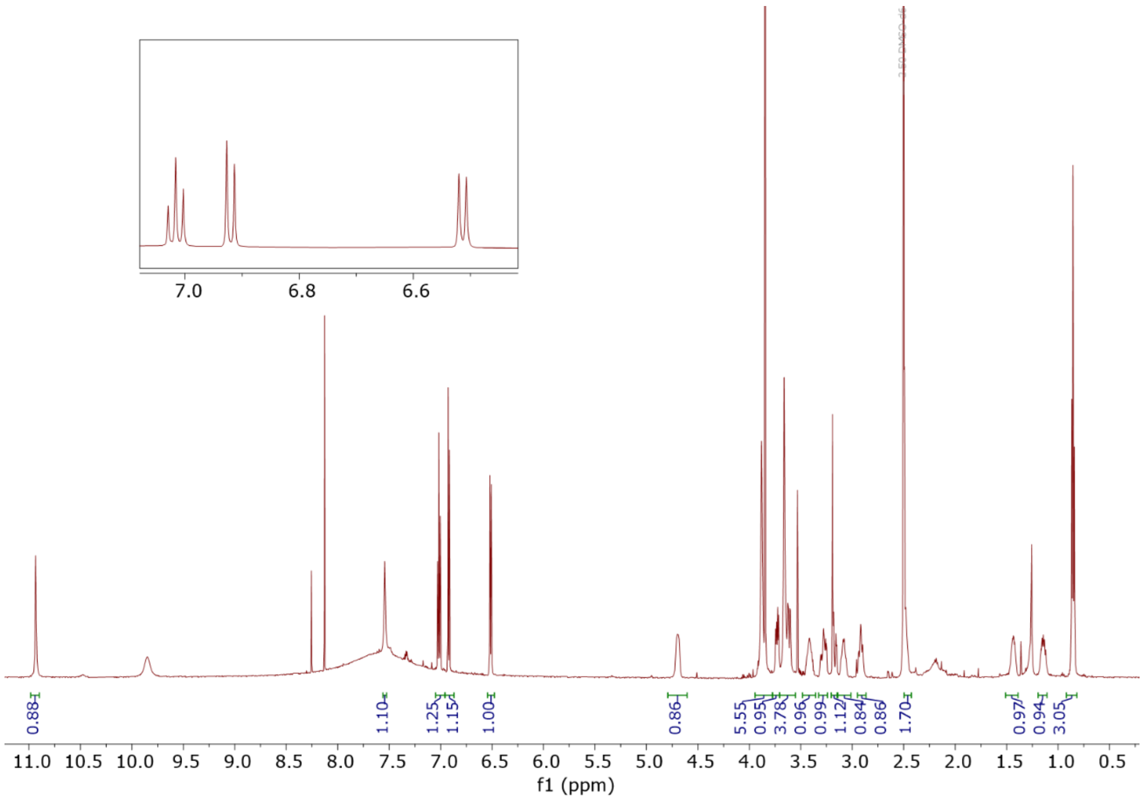

Figure S8. ^{1}H NMR spectrum of speciogynine in DMSO-d_6.

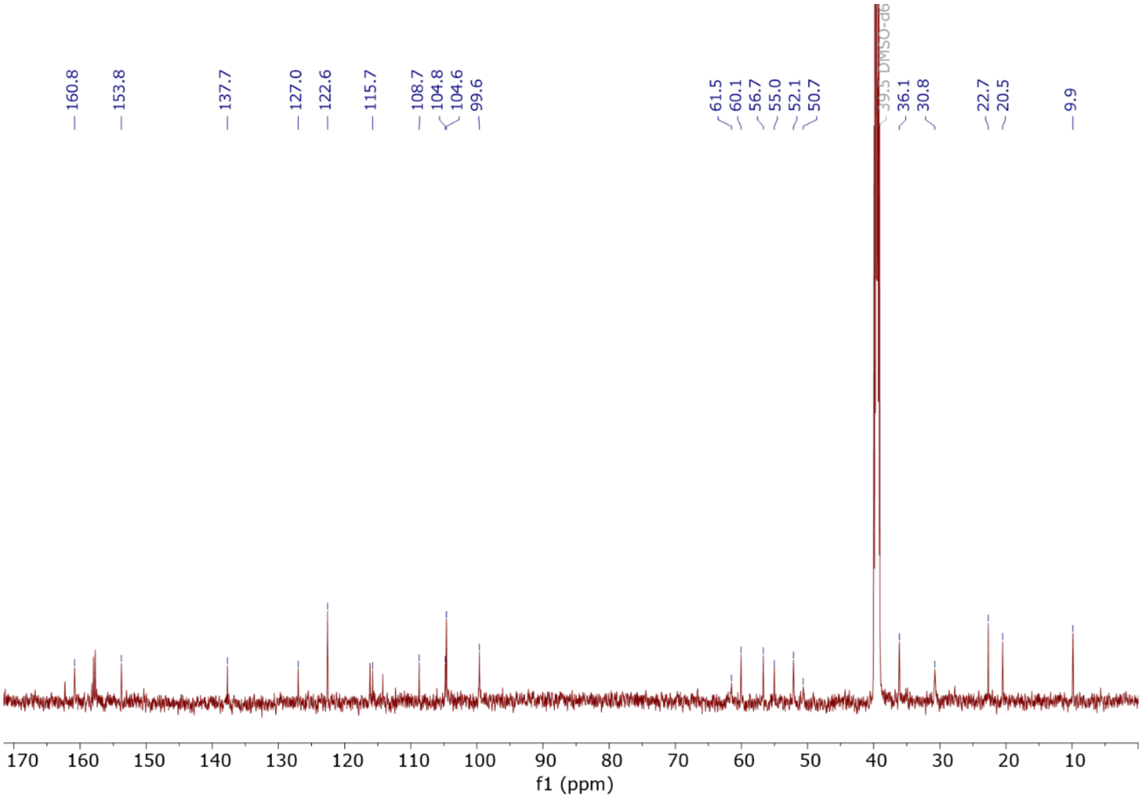

Figure S9. ^{13}C NMR spectrum of speciogynine in DMSO-d_6.

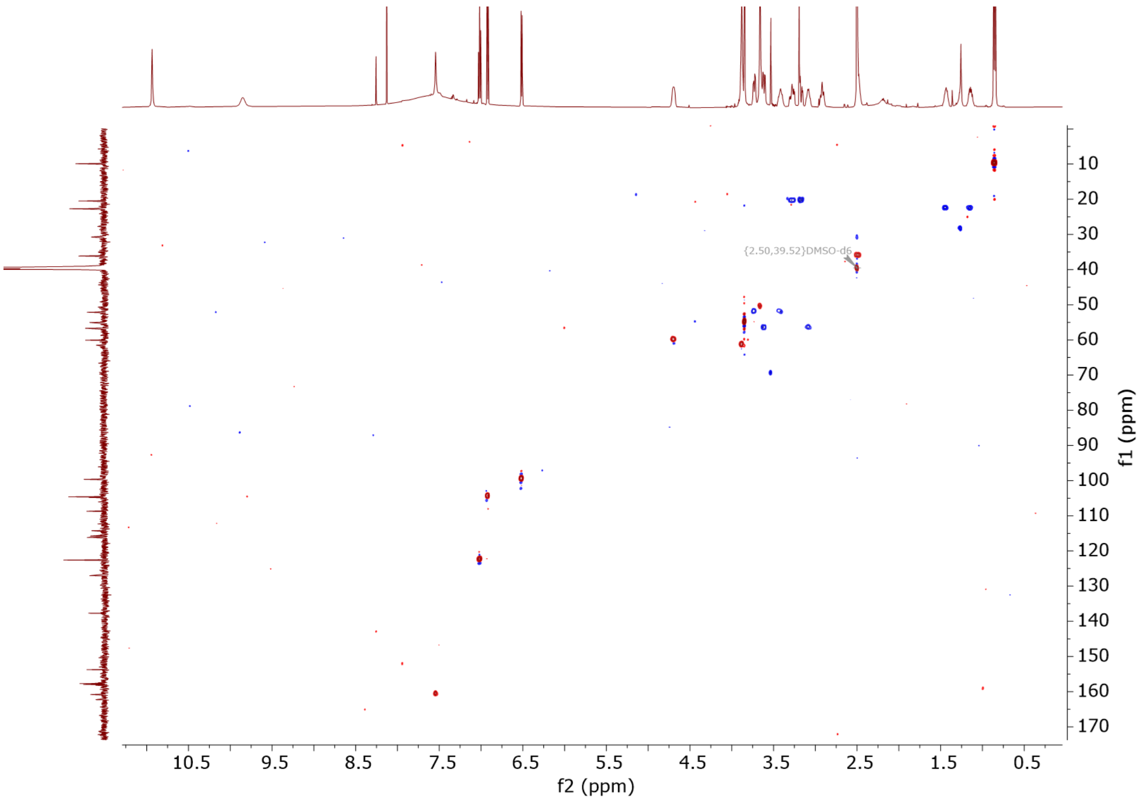

Figure S10. HSQC spectrum of speciogynine in DMSO-*d*6.

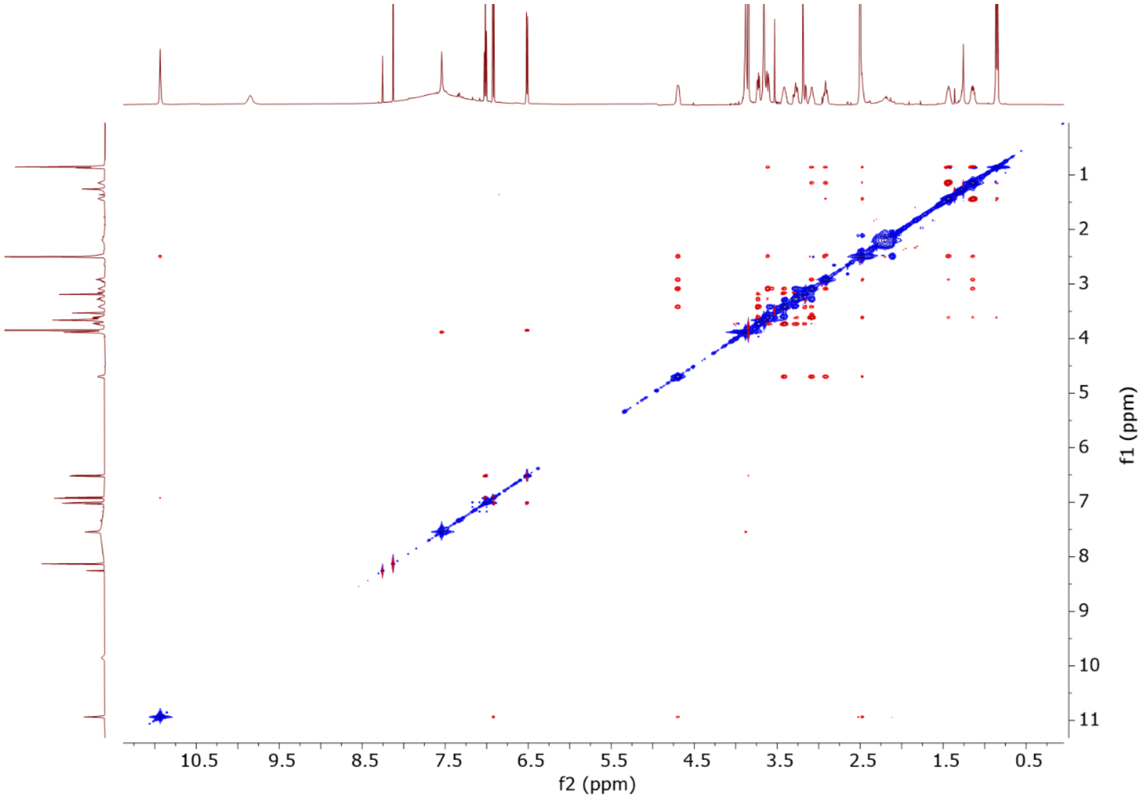

Figure S11. NOESY spectrum of speciogynine in DMSO-d_6.

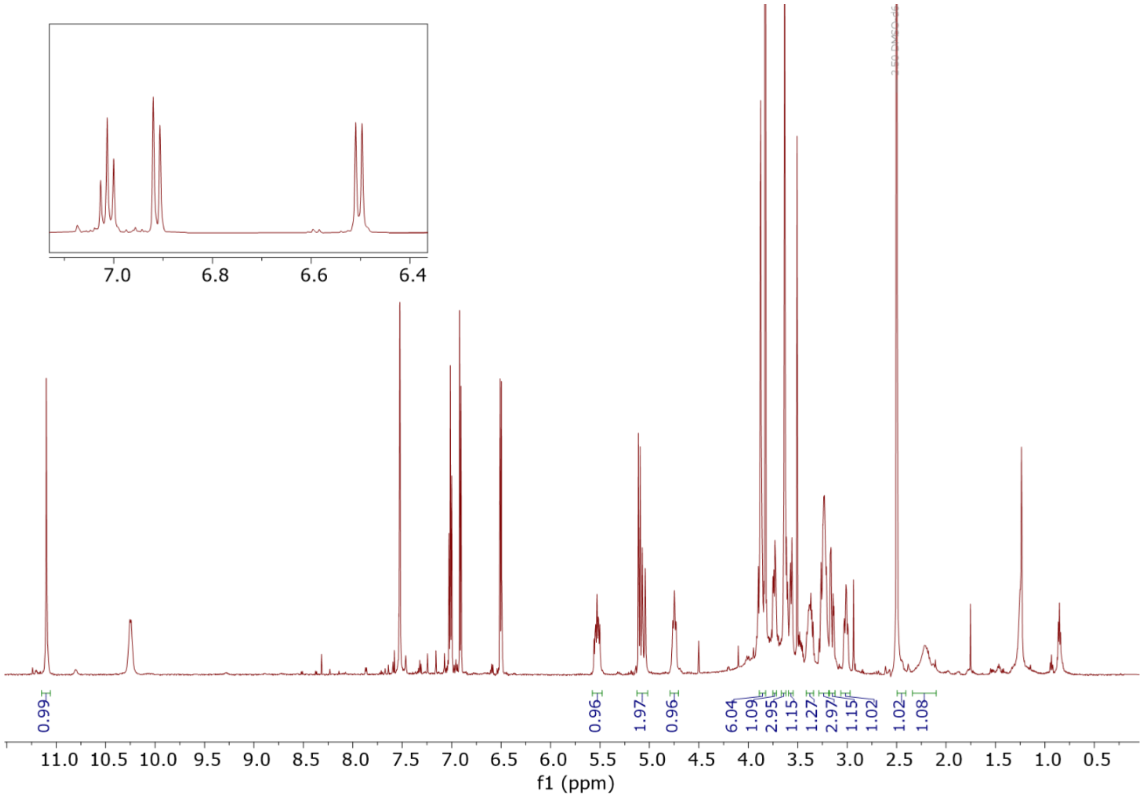

Figure S12. ^{1}H NMR spectrum of paynantheine in DMSO-d_6.

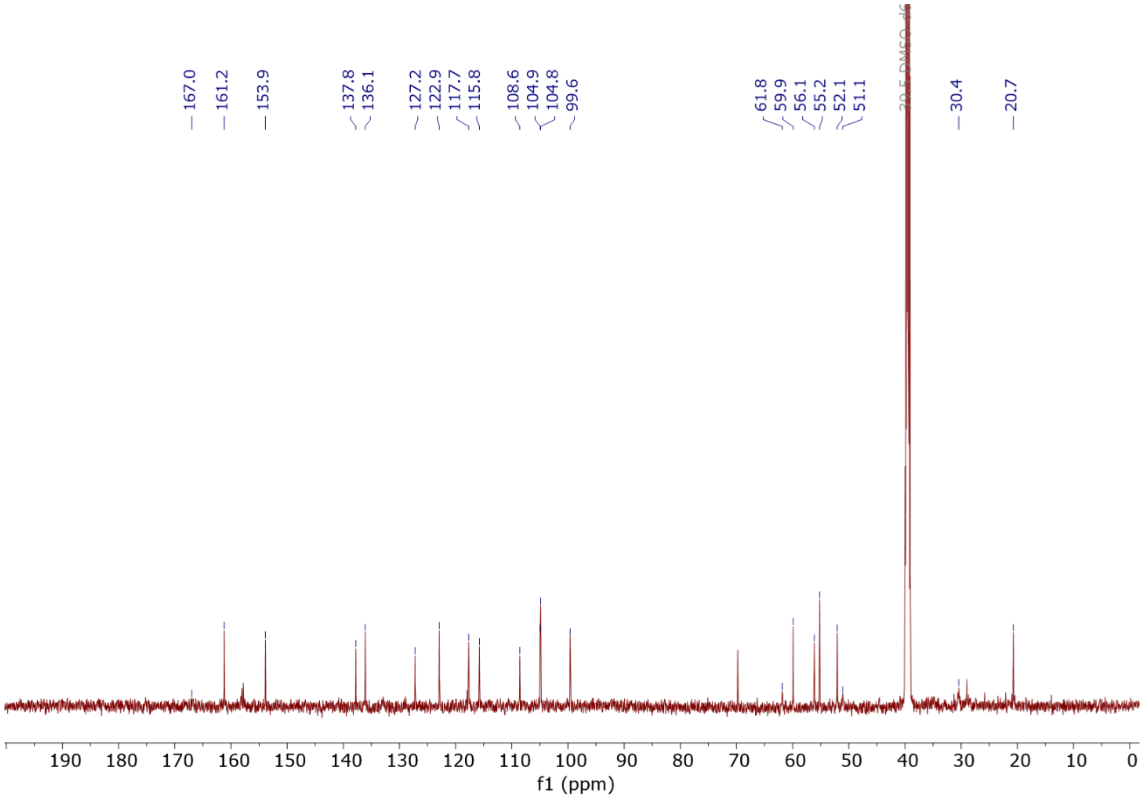

Figure S13. ^{13}C NMR spectrum of paynantheine in DMSO-d_6.

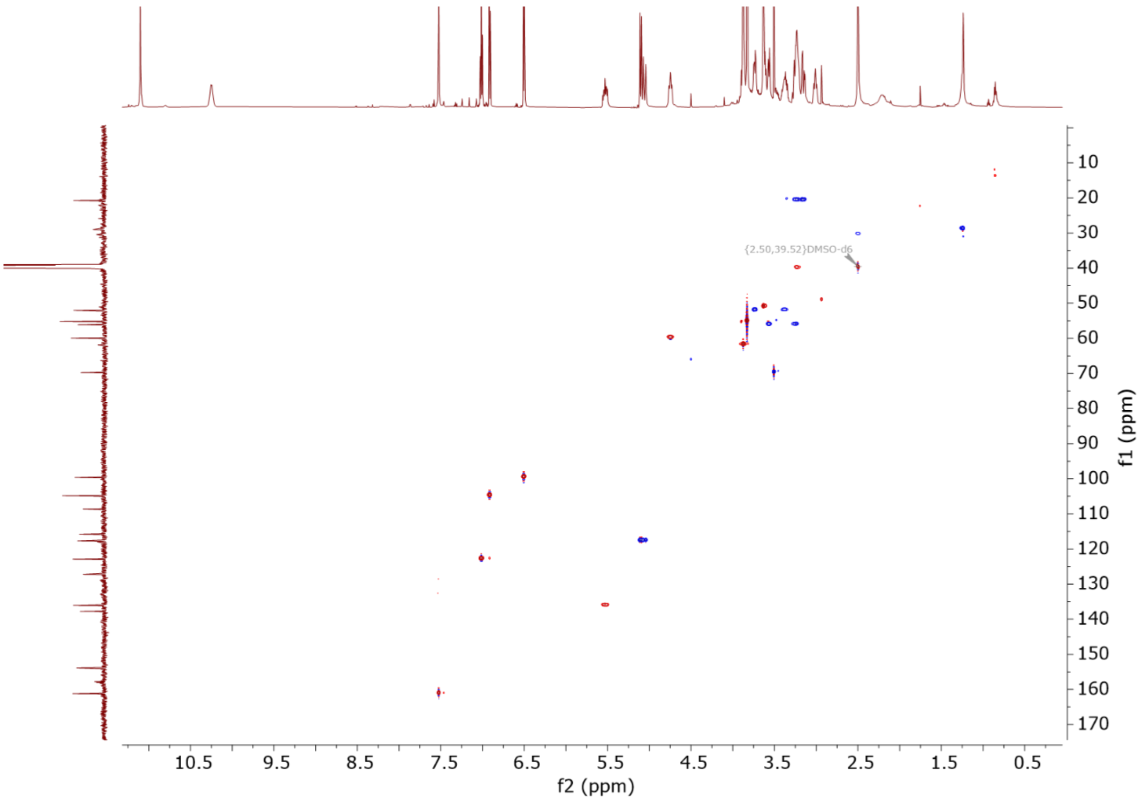

Figure S14. HSQC spectrum of paynantheine in DMSO-d_6.

Table S5. Alkaloid-CD complex stability constants (M^{-1}) measured by affinity capillary electrophoresis at 30 mM phosphate buffer (pH 7.4), 25°C, 15 kV, 215 nm. Further conditions and CD abbreviations can be found in *3.7. Affinity Capillary Electrophoresis* and *3.1. Materials* section.

	Cyclodextrin		Mitragynine	Speciogynine	Speciociliatine	Paynantheine
native CDs	α-CD	K_{stab}	25 ± 2	23 ± 5	22 ± 3	20 ± 2
		μ_{AS}	3.3 ± 0.4	1.8 ± 1.2	2.5 ± 0.9	0.6 ± 0.6
	β-CD	K_{stab}	145 ± 20	65 ± 9	35 ± 3	145 ± 15
		μ_{AS}	6.4 ± 0.3	1.0 ± 0.9	2.7 ± 0.7	0.9 ± 0.4
	γ-CD	K_{stab}	55 ± 5	45 ± 4	15 ± 1	140 ± 5
		μ_{AS}	0.7 ± 0.4	0.5 ± 0.4	0.6 ± 0.3	0.3 ± 0.2
neutral CD derivatives	DIME-β-CD	K_{stab}	150 ± 15	n.d.	100 ± 6	325 ± 30
		μ_{AS}	0.1 ± 0.3	n.d.	2.6 ± 0.2	0.3 ± 0.2
	RAME-β-CD	K_{stab}	95 ± 9	40 ± 1	20 ± 2	170 ± 30
		μ_{AS}	1.4 ± 0.3	2.7 ± 0.1	2.0 ± 0.8	0.2 ± 0.4
	HP-β-CD	K_{stab}	35 ± 2	n.d.	30 ± 3	75 ± 5
		μ_{AS}	0.5 ± 0.3	n.d.	1.5 ± 0.5	0.4 ± 0.2
	HP-γ-CD	K_{stab}	230 ± 35	n.d.	220 ± 25	325 ± 50
		μ_{AS}	5.5 ± 0.3	n.d.	0.6 ± 0.3	0.8 ± 0.3
negatively charged CD derivatives	CM-α-CD	K_{stab}	155 ± 15	135 ± 12	110 ± 10	175 ± 12
		μ_{AS}	-16.0 ± 1.0	-24.7 ± 1.4	-20.4 ± 1.5	-18.8 ± 0.7
	CM-β-CD	K_{stab}	215 ± 20	170 ± 15	230 ± 20	185 ± 15
		μ_{AS}	-7.1 ± 0.6	-10.1 ± 0.7	-5.4 ± 0.6	-13.5 ± 0.7
	CM-γ-CD	K_{stab}	200 ± 10	140 ± 12	120 ± 12	180 ± 20
		μ_{AS}	-11.3 ± 0.4	-13.2 ± 0.8	-10.5 ± 1.1	-16.8± 1.2
	CE-β-CD	K_{stab}	210 ± 20	375 ± 25	160 ± 10	625 ± 40
		μ_{AS}	-10.6 ± 0.7	-8.8 ± 0.5	-13.1 ± 0.7	-13.8 ± 0.3
	Sualfadex	K_{stab}	200 ± 25	75 ± 20	250 ± 45	80 ± 15
		μ_{AS}	-18.1 ± 2.5	-59.0 ± 19.0	-13.1 ± 3.0	-47.5 ± 9.8
	Subetadex	K_{stab}	1 800 ± 80	n.d.	2 700 ± 100	3 600 ± 300
		μ_{AS}	-28.0 ± 0.6	n.d.	-27.4 ± 0.4	-29.3 ± 0.6
	Sugammadex	K_{stab}	2 050 ± 125	n.d.	700 ± 45	2 750 ± 175
		μ_{AS}	-28.5 ± 0.7	n.d.	-49.9 ± 2.3	-31.5 ± 0.6
	Succ-β-CD	K_{stab}	375± 20	840 ± 80	470 ± 35	360 ± 30
		μ_{AS}	-33.2 ± 1.0	-25.8 ± 1.0	-33.5 ± 1.0	-33.4 ± 1.3
	Phos-β-CD	K_{stab}	290 ± 30	420 ± 30	215 ± 15	335 ± 30
		μ_{AS}	-22.6 ± 1.1	-19.8 ± 0.5	-17.5 ± 0.7	-24.2 ± 1.1
	SBE-β-CD DS~4	K_{stab}	550 ± 35	610 ± 60	670 ± 60	830 ± 50
		μ_{AS}	-18.8 ± 0.7	-21.5 ± 0.9	-17.7 ± 0.8	-20.1 ± 0.5
	SBE-β-CD DS~6.5	K_{stab}	1 530 ± 55	1 800 ± 100	1 700 ± 75	2 000 ± 75
		μ_{AS}	-23.7 ± 0.2	-25.1 ± 0.3	-24.3 ± 0.2	-24.5 ± 0.2
	SBE-β-CD DS~10.4	K_{stab}	4 900 ± 420	9 200 ± 890	5 530 ± 435	5 300 ± 280
		μ_{AS}	-32.5 ± 0.4	-32.6 ± 0.3	-32.5 ± 0.4	-33.9 ± 0.3
	SP-β-CD	K_{stab}	210 ± 25	n.d.	n.d.	335 ± 35
		μ_{AS}	-18.3 ± 1.2	n.d.	n.d.	-10.3 ± 0.6
	S-β-CD	K_{stab}	1 650 ± 30	970 ± 80	2 410 ± 185	1 420 ± 145
		μ_{AS}	-22.3 ± 0.2	-23.9 ± 0.9	-17.0 ± 0.6	-24.6 ± 1.2

	**S-γ-CD**	K_{stab}	810 ± 75	n.d.	1 230 ± 140	1 370 ± 145
		μ_{AS}	-36.7 ± 1.7	n.d.	-35.5 ± 2.1	-35.4 ± 1.7
	**HS-β-CD**	K_{stab}	1 250 ± 105	645 ± 30	850 ± 30	575 ± 35
		μ_{AS}	-35.2 ± 1.2	-32.7 ± 0.7	-35.1 ± 0.5	-30.8 ± 0.8
	**HDAS-β-CD**	K_{stab}	4 100 ± 180	2 350 ± 115	3 000 ± 275	1 720 ± 125
		μ_{AS}	-31.7 ± 0.3	-29.2 ± 0.5	-30.3 ± 0.8	-30.4 ± 0.7
	**HxDMS-α-CD**	K_{stab}	50 ± 10	40 ± 4	240 ± 20	110 ± 8
		μ_{AS}	-32.8 ± 8.1	-54.0 ± 5.2	0.4 ± 0.5	-13.6 ± 1.0
	**HDMS-β-CD**	K_{stab}	175 ± 7	30 ± 3	105 ± 12	135 ± 14
		μ_{AS}	-5.8 ± 0.4	-53.6 ± 5.7	-12.4 ± 1.9	-2.7 ± 0.8
	**ODMS-γ-CD**	K_{stab}	80 ± 7	250 ± 15	230 ± 15	280 ± 25
		μ_{AS}	-26.7 ± 2.4	-13.8 ± 0.6	-5.1 ± 0.6	-7.2 ± 0.4
	**HMDiSu-β-CD**	K_{stab}	230 ± 25	530 ± 10	100 ± 1	350 ± 10
		μ_{AS}	-4.0 ± 1.0	-2.6 ± 0.1	-17.8 ± 0.2	-2.8 ± 0.2
**positively charged CD derivatives**	**MA-β-CD**	K_{stab}	< 10	< 10	< 10	< 10
		μ_{AS}	n.d.	n.d.	n.d.	n.d.
	**HPA-β-CD**	K_{stab}	< 10	< 10	< 10	< 10
		μ_{AS}	n.d.	n.d.	n.d.	n.d.
	**PYR-β-CD**	K_{stab}	< 10	< 10	< 10	< 10
		μ_{AS}	n.d.	n.d.	n.d.	n.d.
	**PIP-β-CD**	K_{stab}	< 10	< 10	< 10	< 10
		μ_{AS}	n.d.	n.d.	n.d.	n.d.
	**MePYR-β-CD**	K_{stab}	< 10	< 10	< 10	< 10
		μ_{AS}	n.d.	n.d.	n.d.	n.d.
	**MePIP-β-CD**	K_{stab}	< 10	< 10	< 10	< 10
		μ_{AS}	n.d.	n.d.	n.d.	n.d.

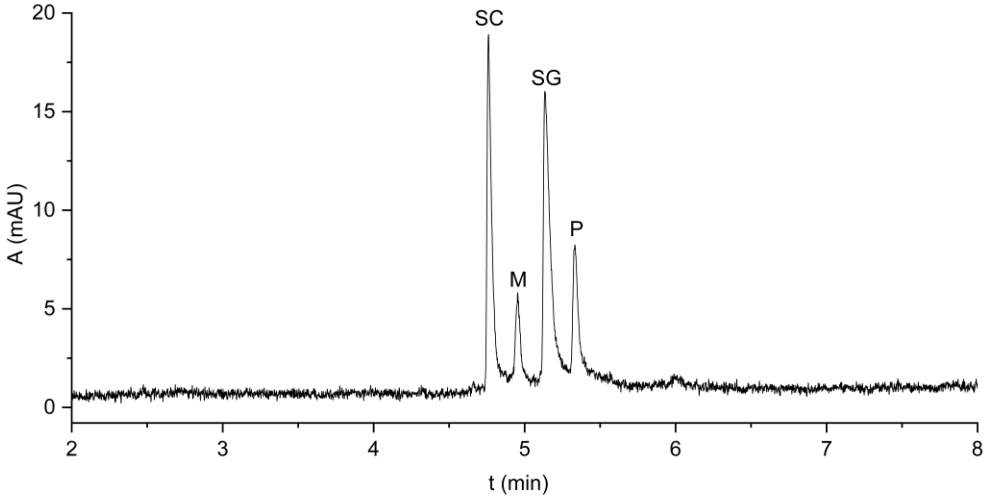

Figure S15: Capillary electrophoretic separation of the four studied alkaloids (SC: speciociliatine, M: mitragynine, SG: speciogynine, P: paynantheine) using 1 mM carboxymethylated-α-cyclodextrin in 30 mM phosphate buffer (pH 7.4), 25°C, 15 kV, 215 nm.

Figure S16: Pilot-scale CPC chromatogram of the preprocessed kratom extract Further conditions

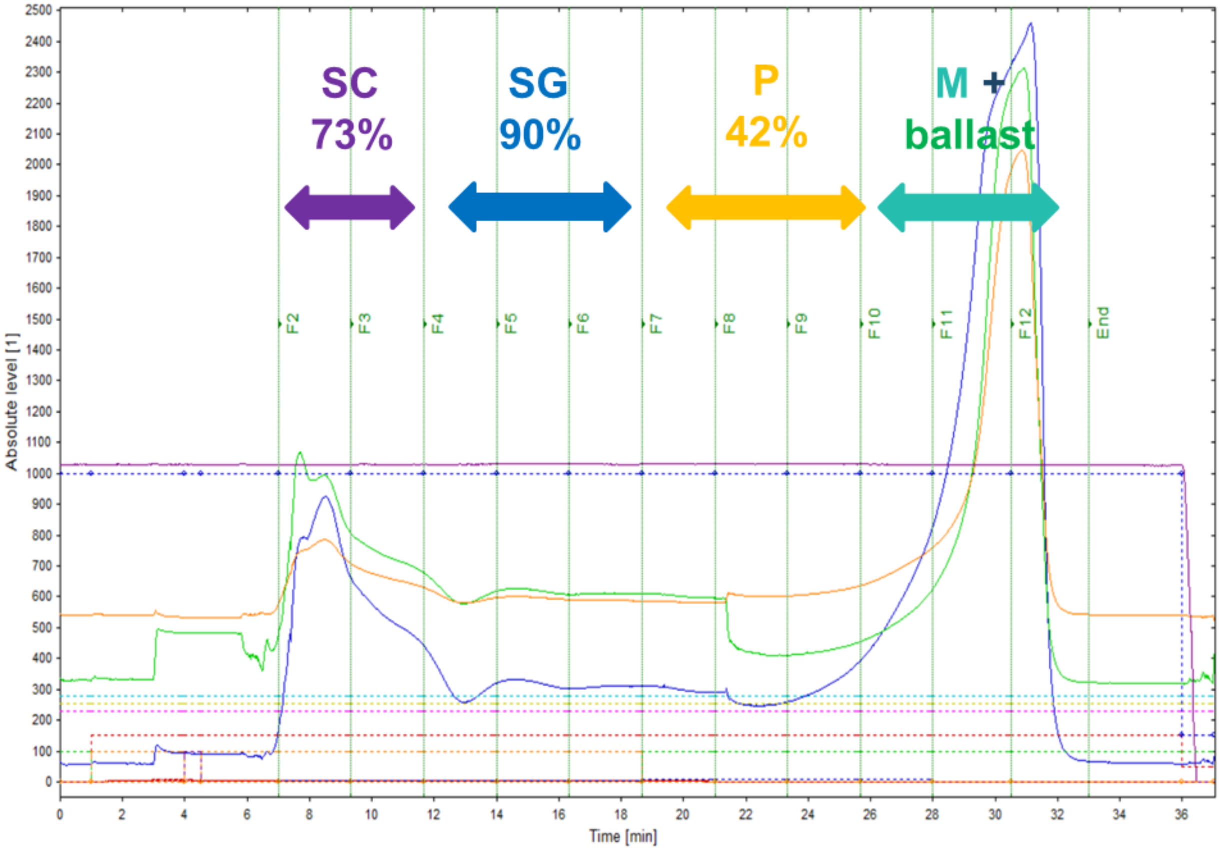

can be found in *3.5. Isolation procedure* section.
